# LncRNA SLCO4A1-AS1 suppresses lung cancer progression by sequestering the TOX4-NTSR1 signaling axis

**DOI:** 10.1186/s12929-023-00973-9

**Published:** 2023-09-19

**Authors:** Yi-Ling Chen, Yi-Nan Liu, Yen-Ting Lin, Meng-Feng Tsai, Shang-Gin Wu, Tzu-Hua Chang, Chia-Lang Hsu, Huey-Dong Wu, Jin-Yuan Shih

**Affiliations:** 1 Graduate Institute of Clinical Medicine, National Taiwan University College of Medicine, Taipei, Taiwan; 2 Department of Internal Medicine, National Taiwan University Hospital, #7, Chung-Shan South Road, Taipei, 100 Taiwan; 3 Department of Medicine, National Taiwan University Cancer Center, Taipei, Taiwan; 4 Department of Biomedical Sciences, Da-Yeh University, Changhua, Taiwan; 5 Department of Medical Research, National Taiwan University Hospital, National Taiwan University, Taipei, Taiwan; 6 Division of Respiratory Therapy, Department of Integrated Diagnostics and Therapeutics, National Taiwan University Hospital, Taipei, Taiwan

**Keywords:** SLCO4A1-AS1, Long non-coding RNA, Cytoskeleton, Metastasis, Lung cancer

## Abstract

**Background:**

Metastasis is a multistep process involving the migration and invasion of cancer cells and is a hallmark of cancer malignancy. Long non-coding RNAs (lncRNAs) play critical roles in the regulation of metastasis. This study aims to elucidate the role of the lncRNA solute carrier organic anion transporter family member 4A1-antisense 1 (SLCO4A1-AS1) in metastasis and its underlying regulatory mechanisms.

**Methods:**

A comprehensive analysis of the Gene Expression Omnibus (GEO) database were used to identify metastasis-associated lncRNAs. Transwell migration and invasion assays, and a tail vein-injection mouse model were used to assess the migration and invasion of cancer cells in vitro and in vivo, respectively. High-throughput screening methods, including MASS Spectrometry and RNA sequencing (RNA-seq), were used to identify the downstream targets of SLCO4A1-AS1. Reverse transcription quantitative polymerase chain reaction (RT-qPCR), western blotting, RNA pull-down, RNA immunoprecipitation (RIP), fluorescence in situ hybridization (FISH), and chromatin immunoprecipitation (ChIp) assays were conducted to identify and validate the underlying regulatory mechanisms of SLCO4A1-AS1.

**Results:**

SLCO4A1-AS1 reduced cancer cell migration and invasion by disrupting cytoskeleton filaments, and was associated with longer overall survival in patients with lung adenocarcinoma. SLCO4A1-AS1 directly interacted with the DNA-binding protein, TOX High Mobility Group Box Family Member 4 (TOX4), to inhibit TOX4-induced migration and invasion. Furthermore, RNA-seq revealed that neurotensin receptor 1 (NTSR1) is a novel and convergent downstream target of SLCO4A1-AS1 and TOX4. Mechanistically, SLCO4A1-AS1 functions as a decoy of TOX4 by interrupting its interaction with the *NTSR1* promoter and preventing *NTSR1* transcription. Functionally, *NTSR1* promotes cancer cell migration and invasion through cytoskeletal remodeling, and knockdown of NTSR1 significantly inhibits TOX4-induced migration and invasion.

**Conclusion:**

These findings demonstrated that SLCO4A1-AS1 antagonizes TOX4/NTSR1 signaling, underscoring its pivotal role in lung cancer cell migration and invasion. These findings hold promise for the development of novel therapeutic strategies targeting the SLCO4A1-AS1/TOX4/NTSR1 axis as a potential avenue for effective therapeutic intervention in lung cancer.

**Supplementary Information:**

The online version contains supplementary material available at 10.1186/s12929-023-00973-9.

## Background

Lung cancer is the leading cause of mortality among malignant tumors [1]. Historically, lung cancer was often diagnosed at advanced stages, owing to the unavailability of early diagnostic strategies. Furthermore, distant metastasis of cancer cells resistant to antitumor therapy accounts for nearly 90% of cancer-related deaths [2]. Despite recent advances in the diagnosis and treatment of lung cancer, metastasis remains a major challenge in advanced lung cancer [3]. Therefore, it is imperative to elucidate new molecular pathways underlying cancer invasion and metastasis for the development of effective therapeutic strategies.

Emerging evidence has shown that long non-coding RNAs (lncRNAs) play critical roles in the development of human diseases, especially cancer [4]. LncRNAs are more than 200 nucleotides in length with no protein-coding capabilities and regulate gene expression through multiple mechanisms. These mechanisms include chromatin remodeling, transcriptional regulation, and post-transcriptional modification of mRNA or protein activity [5, 6]. Recent studies have reported that lncRNAs may act as oncogenic drivers or tumor suppressors, regulating the migration, invasion, and metastasis of lung cancer by modulating gene regulation during cancer progression [7]. LncRNAs act as competing endogenous RNA (ceRNAs) that sponge certain miRNAs to indirectly regulate downstream gene expression. For example, MALAT1 recruits miR-200a-3p and miR-204 to counteract the suppression of programmed death ligand 1 (PD-L1) and the zinc-finger transcription repressor, Slug [8, 9]. This promotes epithelial-mesenchymal transition (EMT), proliferation, and metastasis in lung adenocarcinoma. Additionally, LIFR-AS1 can function as a sponge for miR-942-5p to inhibit metastasis in non-small cell lung cancer (NSCLC) by suppressing ZNF471 expression [10].

LncRNAs also influence lung cancer metastasis through various distinct signaling pathways. For instance, the Wnt/β-catenin signaling pathway, crucial for tumor growth, invasion, and metastasis, is affected by lncRNA BCYRN1 in NSCLC [11]. LncRNA BCYRN1 upregulation leads to reduced cyclinD1 expression and activates Wnt/β-catenin signaling, promoting cell proliferation and metastasis in NSCLC [12]. The PI3K/AKT pathway, integral to cancer progression, metastasis, and metabolism, is influenced by lncRNAs such as TM4SF1-AS1, HOXB-AS3, and TP73-AS1, promoting lung cancer cell migration and invasion [13–16]. Conversely, LncFOXO1 inhibits lung cancer cell proliferation and metastasis while inducing apoptosis by attenuating PI3K/AKT signaling [17]. Moreover, several lncRNAs interact with EZH2, a histone lysine methyltransferase subunit of polycomb repressive complex 2 (PRC2), to regulate lung cancer metastasis. EZH2 catalyzes the methylation of H3K27, play a crucial role in epigenetic gene silencing [18]. LncRNAs UFC1 and SNHG20 bind to EZH2 to inhibit the expression of PTEN and p21, respectively, which promotes the metastasis of NSCLC [19, 20]. Thus, the biogenesis and regulatory mechanisms of lncRNAs provide valuable insights into tumor biology and present promising therapeutic targets for advanced NSCLC.

The lncRNA solute carrier organic anion transporter family member 4A1-antisense 1 (SLCO4A1-AS1) plays an oncogenic role in various cancer types. SLCO4A1-AS1 acts as a sponge or scaffold to modulate downstream proteins and signaling pathways. SLCO4A1-AS1 functions as a molecular scaffold to enhances the interaction between Hsp90 and Cdk2, resulting in enhanced stability of Cdk2 [21]. Consequently, the increase in Cdk2 levels activates the c-Myc signaling pathway, resulting in increased tumor growth in colorectal cancer. Most studies have found that SLCO4A1-AS1 functions as a miRNA sponge and is recognized as a competing RNA. SLCO4A1-AS1 recruits miRNAs to disrupt their interaction with downstream target mRNAs that promote tumorigenesis and tumor progression. For example, in lung cancer, it increases IKKα expression by competitively binding with miR-223-3p, which consequently activates the NF-κB signaling pathway [22]. Similarly, in colorectal cancer, SLCO4A1-AS1 promotes cell proliferation by regulating autophagy through decoying miR-508-3p, leading to the upregulation of PARD3 [23]. Additionally, SLCO4A1-AS1 directly binds to β-catenin, thereby stabilizing it by preventing interaction with GSKβ and phosphorylation. This activates the Wnt/β-catenin signaling pathway, leading to an increase in proliferation, migration, and invasion in colorectal cancer [24]. Nevertheless, the specific role of SLCO4A1-AS1 in NSCLC remains unclear.

In this study, we identified candidate lncRNAs associated with the metastatic potential of lung cancer cells by analyzing differential gene expression profiles of highly metastatic PC9/gef cells and corresponding low-metastatic PC9 cells from the Gene Expression Omnibus (GEO) database (GSE60189). We found that the SLCO4A1-AS1 (NR_024470) was downregulated in highly metastatic cells and explored its role in regulating the migration and invasion of lung cancer cells. Furthermore, we investigated the interactions between SLCO4A1-AS1 and the downstream TOX High Mobility Group Box Family Member 4 (TOX4) and neurotensin receptor 1 (NTSR1) pathways. Our findings indicate that the SLCO4A1-AS1/TOX4/NTSR1 axis may be a potential therapeutic target for NSCLC.

## Methods

### Cell lines and cell culture

The human cancer cell line PC9 and its derivative cell line PC9/gef were provided by Dr. James Chih-Hsin Yang (National Taiwan University Hospital) [25]. CL1-0 and CL1-5 were obtained from Dr. Pan-Chyr Yang (National Taiwan University Hospital, Taiwan). H1299 and A549 cells were purchased from American Type Culture Collection (ATCC; Manassas, VA, USA). All cell lines were cultured in RPMI-1640 (Thermo Fisher Scientific, Waltham, MA, USA) supplemented with 10% fetal bovine serum (FBS; Thermo Fisher Scientific) and penicillin-streptomycin-amphotericin B solution (Biological Industries, Beit Haemek, Israel). Cells were cultured at 37 °C in a humidified atmosphere containing 5% CO_2_. All cell lines were authenticated using short tandem repeat (STR) profiling and tested negative for mycoplasma contamination.

### Microarray analysis

Gene expression datasets for PC9/gef and PC9 cells were downloaded from the NCBI Gene Expression Omnibus (GEO) database (accession number GSE60189). Raw files were processed and normalized using the RMA package and differential expression analysis was performed using the limma package. Probes with a q-value < 0.01 and fold-change > 2 were considered as differentially expressed genes. Significant lncRNAs were identified using heat maps generated using GraphPad Prism 8 software.

### RNA sequencing (RNA-seq) and bioinformatics analysis

Total RNA from two pairs of cell lines (H1299-mock vs. H1299-SLCO4A1-AS1; H1299-mcok vs. H1299-TOX4) was isolated, and the sequencing core of the Medical Research Department of National Taiwan University Hospital performed subsequent analyses. This included sample quality control, library construction, and RNA sequencing. Library construction was performed using the SureSelect Strand-Specific RNA Library kit (Agilent, Santa Clara, CA, USA) and RNA-seq was performed using Illumina MiniSeq (Illumina). Read counts for individual datasets were normalized using the trimmed mean of M-values (TMM) method in the edgeR package and converted to log_2_ counts per million (logCPM). Criteria for selecting differentially expressed genes (DEGs) were |log_2_ fold change (FC)| > 1.5 and adjusted *p* value < 0.05. We used the Database for Annotation, Visualization, and Integrated Discovery (DAVID version 6.8) to elucidate potential gene ontology (GO) functions [26].

### Real-time quantitative PCR

Total RNA was extracted using Tri reagent (Molecular Research Center, Cincinnati, OH, USA) and cDNA synthesis was performed using the SuperScript™ IV Reverse Transcriptase Kit (Thermo Fisher Scientific). Quantitative PCR was performed on a QuantStudio™ 7 Flex Real-Time PCR System following a standard protocol, with TATA box-binding protein (TBP) as an internal control. The relative mRNA expression levels were calculated using the 2^−ΔCt^ method, where ΔCt = (sample Ct − TBP Ct). Ct values > 40 were considered “undetectable”. The sequences of gene-specific primers are listed in Additional file 1: Table S1.

### Construction of stable clones

Full-length SLCO4A1-AS1 (NR_024470; LNCipedia transcript ID: SLCO4A1-AS1:4; https://lncipedia.org/) was synthesized and subcloned into the pLAS2w.pPuro vector (Academia Sinica, Taipei, Taiwan) [27]. Human TOX4 and NTSR1 ORF cDNA clones were purchased from Sino Biological (Beijing, China). The full-length TOX4 sequence was cleaved and re-ligated into the pLAS3w.pPneo vector (Academia Sinica) using restriction enzymes. Following transfection of 293T cells with respective vectors (pLAS2w.pPuro-SLCO4A1-AS1 or pLAS3w.pPneo-TOX4, pMD.G and pCMVΔ8.91), viral particles were harvested for the infection of the lung cancer cell lines.

### RNA interference

Gene knockdown was achieved by transfecting cells with small interfering RNAs (siRNAs) using Lipofectamine RNAiMax reagent (Thermo Fisher Scientific), according to the manufacturer’s instructions. siRNAs targeting human SLCO4A1-AS1 (#R-187800-00-0010) were purchased from Horizon Discovery (Cambridge, UK). siRNAs targeting human TOX4 (#S19128; human TOX4-siRNA) and NTSR1 (#S9767; human NTSR1-siRNA) were obtained from Thermo Fisher Scientific.

### Western blotting

Cells were lysed using RIPA buffer (Cell Signaling Technology, Danvers, MA, USA). Proteins were quantified and resolved using sodium dodecyl sulfate-polyacrylamide gel electrophoresis, transferred to PVDF membranes, and incubated with the indicated primary antibodies. The blots were then incubated with horseradish peroxidase (HRP)-linked secondary antibodies and immunoreactive bands were visualized using enhanced chemiluminescence reagents (Merck Millipore, Burlington, MA, USA). The antibodies used in this study are listed in Additional file 2: Table S2.

### In vitro migration and invasion assay

The cells were harvested and resuspended in serum-free medium. For the migration assay, cells were plated on Transwell inserts and 5% FBS was used as a chemoattractant. For the invasion assay, the cells were seeded into a pre-packed Matrigel chamber insert (#354480; Corning Life Sciences, Oneonta, NY, USA). After 18–24 h of incubation, the cells remaining on the upper membrane surface were removed using a cotton swab, and the cells adhering to the lower membrane surface were fixed with methanol and stained with 0.1% crystal violet. Cells were counted using an optical microscope.

### Biotin-labeled SLCO4A1-AS1, RNA pull-down, and RNA immunoprecipitation (RIP) assays

In the RNA pull-down assay, RNAs were synthesized through in vitro transcription with T7 RNA polymerase using the MEGAscript™ T7 Transcription Kit (Thermo Fisher Scientific). Subsequently, the RNA was biotin-labeled using the Pierce™ RNA 3′End Desthionbiotinylation Kit (Thermo Fisher Scientific). RNA-protein pull-down was performed using the Pierce™ Magnetic RNA-Protein Pull-Down Kit (Thermo Fisher Scientific). Briefly, labeled RNA was captured using streptavidin magnetic beads. Cell lysates were mixed and incubated with biotinylated RNA, followed by incubation for 1 h at 4 °C with rotation. The beads were washed and boiled in SDS buffer, and the retrieved proteins were detected by western blot analysis. RIP experiments were performed using the Dynabeads™ Protein A/G Immunoprecipitation Kit (Thermo Fisher Scientific) according to the manufacturer’s instructions. The co-precipitated RNA was detected using RT-qPCR.

### Chromatin immunoprecipitation (ChIP) assay

For ChIP assays, the Pierce™ Magnetic ChIP Kit (Thermo Fisher Scientific) was used, in accordance with the manufacturer’s instructions. Cells were fixed with 1% formaldehyde and crosslinking was terminated by the addition of 0.125 M glycine. Subsequently, cells were harvested by scraping and the resulting cell pellets were disrupted. Nuclei were collected using a membrane extraction buffer, digested with micrococcal nuclease (MNase), and lysed by sonication to obtain chromatin. The chromatin complex was incubated with TOX4 antibody overnight at 4 °C. Protein A/G magnetic beads were added to the antibody-chromatin complex and mixed for 2 h. RNase A and proteinase K were used to reverse crosslink the chromatin samples. The DNA was purified and subjected to ChIP-qPCR. The specific primers for the promoter sequences of *NTSR1* are listed in Additional file 1: Table S1.

### Immunofluorescence staining

Cells were seeded onto chamber slips and left to adhere overnight. Subsequently, they were fixed with 4% paraformaldehyde and incubated with primary antibodies targeting p-FAK (#44-625G, Thermo Fisher Scientific) and p-paxillin (#ab32115, Abcam, Cambridge, UK). Fluorescence-conjugated F-actin probe rhodamine phalloidin (#A34055, Thermo Fisher Scientific) was used to stain and visualize the actin filaments. Phospho-FAK and phospho-paxillin were visualized using fluorescence-conjugated secondary antibodies (#A-21428, Thermo Fisher Scientific). 4′,6-Diamidino-2-phenylindole (DAPI) was used for nuclear staining. Images were captured using a ZEISS LSM 880 confocal microscope and analyzed using ZEN 2.3 SP1 (black) software. The antibodies used for immunofluorescence staining are listed in Additional file 2: Table S2.

### RNA fluorescence in situ hybridization (FISH)

RNA-FISH was performed using an RNAscope® Fluorescent Multiplex Reagent Kit (#320850; Advanced Cell Diagnostics (ACD), Newark, CA, USA). Briefly, the cells were seeded into chamber slides in growth medium a day before fixation. The cells were fixed with 10% neutral-buffered formalin for 30 min at room temperature. After fixation, standard RNAscope protocols were adhered to in accordance with the manufacturer’s instructions. For the detection of lncRNA SLCO4A1-AS1, cells were hybridized with the SLCO4A1-AS1-specific RNAscope probe (NPR-0007887, ACD) using the HybEZ Hybridization System at 40 °C for 2 h. The RNAScope positive control probe, Hs-PPIB (#323901, ACD), was used in this study. Following hybridization, amplification and detection were carried out using the RNAscope® Fluorescent Multiplex Reagent Kit according to the manufacturer’s instructions. Images were captured using a ZEISS LSM 880 confocal microscope and analyzed using the ZEN 2.3 SP1 (black) software.

### Tail vein metastasis assay

All animal experiments were conducted in accordance with relevant guidelines and approved by the Institutional Animal Care and Use Committee (IACUC) of the National Taiwan University College of Medicine. Four-week-old male NOD/SCID mice were purchased from BioLASCO Taiwan Co., Ltd. and bred under specific pathogen-free conditions at the Laboratory Animal Center, College of Medicine, National Taiwan University. H1299 cells (2 × 10^6^) stably expressing SLCO4A1-AS1, TOX4, or the control vector in 150 µL of Hanks’ balanced salt solution (HBSS) were injected into the tail vein of the mice. After seven weeks, the mice were humanely euthanized using carbon dioxide anesthesia, and their lung tissues were excised for further analysis.

### Statistical analysis

Statistical analyses were performed using GraphPad Prism 8 and Excel. The results are expressed as the mean ± standard deviation (SD), and all experiments were performed in triplicate. The Student’s t-test was used to compare differences between groups. The paired Wilcoxon test was used to compare the expression level between paired tumor and adjacent normal tissue. For all analyses, *p*-values < 0.05 from two-tailed tests were considered statistically significant.

## Results

### SLCO4A1-AS1 expression is downregulated in highly metastatic lung cancer cells and NSCLC patients

**Fig. 1:**
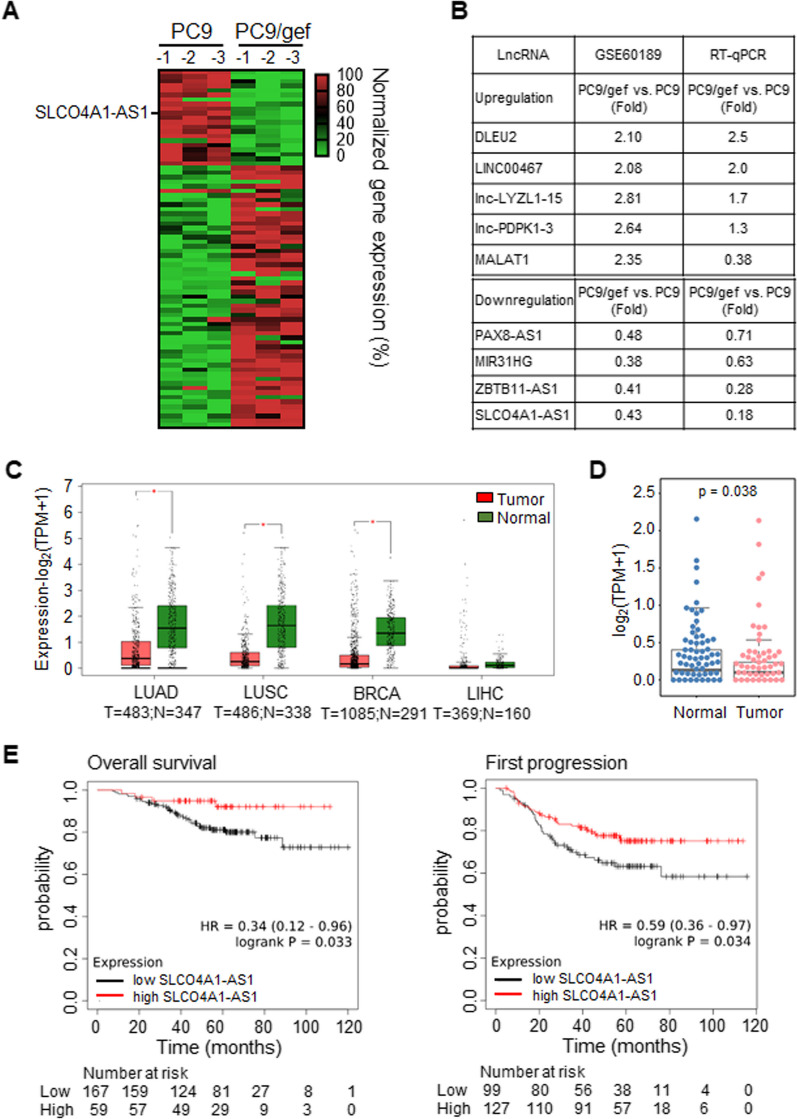
SLCO4A1-AS1 is significantly downregulated in highly metastatic lung cancer cells and NSCLC patients.** A** Heat map of lncRNA expression in PC9 and PC9/gef cells from the GEO database (GSE60189). **B** The differential expression fold change of lncRNAs is listed from the database (left) and validated by RT-qPCR (right). **C** SLCO4A1-AS1 expression in LUAD, LUSC, BRCA, and LIHC tissues from Gene Expression Profiling Interactive Analysis (GEPIA) database. **D** RNA-seq data revealed SLCO4A1-AS1 transcript expression in 90 pairs of lung tumors and corresponding adjacent normal tissues in Taiwan Lung Adenocarcinoma Patient Cohort (dbGaP Study Accession: phs001954.v1.p1). The data was analyzed using a paired Wilcoxon test *(p* = 0.038). **E** The correlation between SLCO4A1-AS1 expression and overall survival (log rank test, *p* = 0.033), and first progression (log rank test, *p* = 0.034) of lung cancer patients from GSE31210 in Kaplan–Meier plotter database website. *LUAD* lung adenocarcinoma, *LUSC* lung squamous cell carcinoma, *BRCA* breast invasive carcinoma, *LIHC* liver hepatocellular carcinoma

We identified candidate lncRNAs that may be associated with metastatic capacity by comparing the gene expression profiles of highly metastatic PC9/gef and low metastatic PC9 cells from the GEO database (GSE60189) and identified 79 differentially expressed lncRNAs (Fig. 1A; Additional file 3: Table S3). Among these, 22 lncRNAs were upregulated and 57 were downregulated in highly metastatic PC9/gef cells compared to those in PC9 cells. Nine lncRNAs were selected for RT-qPCR analysis (Fig. 1B). Among them, four upregulated (DLEU2, LINC00467, lnc-LYZL1-15, and lnc-PDPK1-3) and four downregulated lncRNAs (PAX8-AS1, MIR31HG, ZBTB-AS1, and SLCO4A1-AS1) were consistent with microarray findings, with the exception of MALAT1 (Fig. 1B). Notably, SLCO4A1-AS1 exhibit the most significant downregulation in PC9/gef cells. Furthermore, we examined SLCO4A1-AS1 expression levels in NSCLC tissues and normal samples using the Gene Expression Profiling Interactive Analysis (GEPIA) database [28]. SLCO4A1-AS1 transcript levels were significantly lower in both lung adenocarcinoma (LUAD) and lung squamous cell carcinoma (LUSC) tissues. Additionally, the expression of SLCO4A1-AS1 was consistently downregulated in both breast invasive carcinoma (BRCA) and liver hepatocellular carcinoma (LIHC) tissues (Fig. 1C). Moreover, we extended our analysis to the Taiwan Lung Adenocarcinoma Patient Cohort (dbGaP Study Accession: phs001954.v1.p1), comprising of 90 pairs of lung tumors and adjacent normal tissues [29]. In this cohort, the expression level of SLCO4A1-AS1 was significant lower in tumor tissues than in the corresponding normal tissues (Fig. 1D). Notably, lung cancer patients with tumors with higher SLCO4A1-AS1 expression had significantly improved overall survival and extended time to recurrence based on Kaplan–Meier plotter analysis (Fig. 1E; https://kmplot.com/analysis/; GSE31210) [30]. Thus, the aberrant downregulation of SLCO4A1-AS1 in lung tumors correlated with clinical outcomes and prognosis.

### Biological process enrichment analysis of SLCO4A1-AS1

**Fig. 2:**
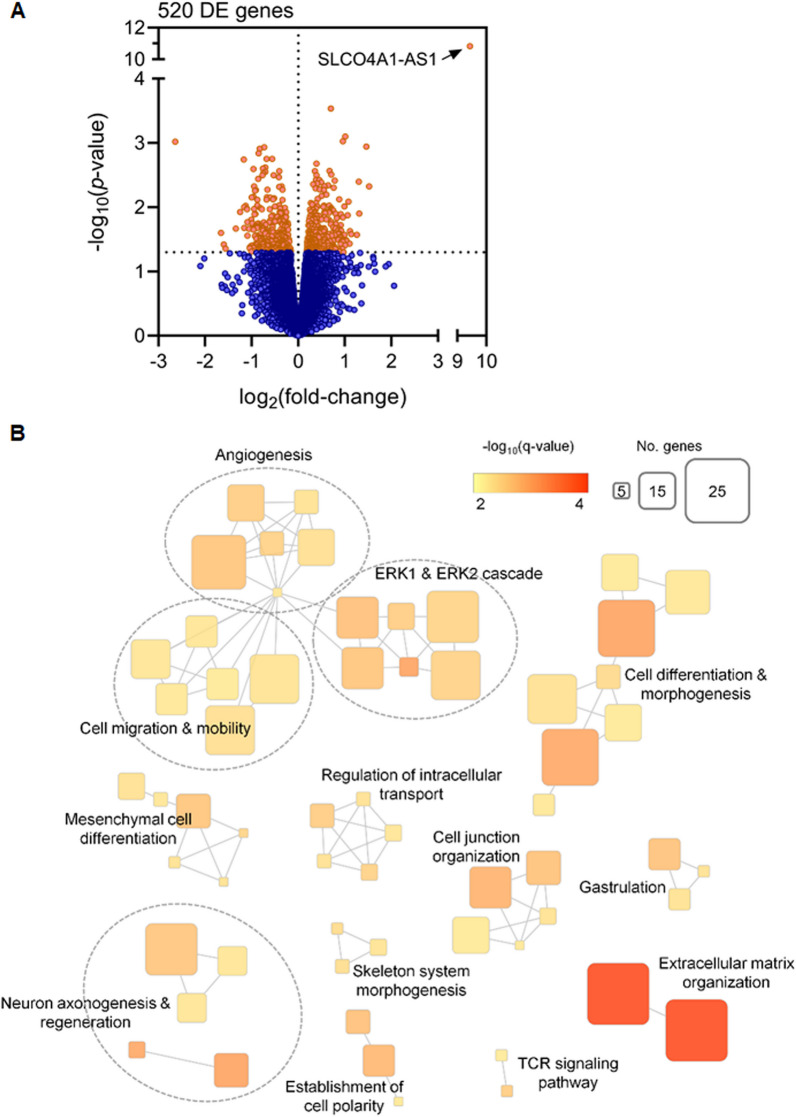
Enrichment analysis for biological process of SLCO4A1-AS1.** A** Volcano plot comparing differential gene expression in H1299-mock vs. H1299-SLCO4A1-AS1 cells. The cutoff was set as |log2(fold change)| > 1.5 and adjusted *p* value < 0.05. **B** Enrichment map of the top 520 SLCO4A1-AS1-related genes. Nodes represent Gene Ontology (GO) terms, node colors and size indicate the enrichment significance and number of GO-associated genes. Edges indicate substantial overlap between GO-related genes

We established H1299-mock and H1299-SLCO4A1-AS1 cells to compare the differentially expressed gene (DEG) profiles of H1299-mock and H1299-SLCO4A1-AS1 cells by performing RNA-seq (Fig. 2A; Additional file 8: Fig. S1A). Using Gene Ontology (GO) enrichment analysis, we categorized 520 DEGs into 15 GO biological processes, including extracellular matrix organization, cell migration and mobility, cell junction organization, and cell differentiation and morphogenesis (Fig. 2B). These results indicated that SLCO4A1-AS1 plays a role in regulating cell adhesion and motility.

### SLCO4A1-AS1 plays a tumor suppressor role in lung cancer cells

We further explored the role of SLCO4A1-AS1 in lung cancer by overexpressing it in lung cancer cell lines (PC9/gef, CL1-5, and H1299) using a plasmid encoding the full-length sequence of the SLCO4A1-AS1 transcript (NR_024470). RT-qPCR confirmed the elevated expression of SLCO4A1-AS1 (Additional file 8: Fig. S1A). We assessed the effects of SLCO4A1-AS1 on various aspects of cancer cell behavior, including proliferation, stemness, sphere formation, drug resistance, invasion, and migration. Sphere-forming assays indicated that SLCO4A1-AS1-overexpressing cells exhibited reduced sphere size and number compared to control cells (Additional file 8: Fig. S1B, C). Increased expression and activity of aldehyde dehydrogenase 1A1 (ALDH1A1) have been reported as robust cancer stem cell markers [31]. SLCO4A1-AS1-overexpression decreased the population of ALDH^+^ cells and *ALDH1A1* mRNA levels compared to control cells (Additional file 8: Fig. S1D, E). Additionally, we used Hoechst 33342 dye and verapamil to characterize the side population, which has been shown to be enriched for cancer stem-like cells. We found a significant decrease in the side population fraction in PC9/gef-SLCO4A1-AS1 cells compared to PC9/gef-mock cells (0.38% vs. 0.20%) and H1299-SLCO4A1-AS1 cells compared to H1299-mock cells (5.80% vs. 4.08%) (Additional file 8: Fig. S1F), which supports a negative correlation between SLCO4A1-AS1 and stem-like characteristics. However, SLCO4A1-AS1-overexpression did not alter cell proliferation or EGFR-TKI/chemotherapeutic agent sensitivity (Additional file 8: Fig. S2A, B). SLCO4A1-AS1 exerted a suppressive effect on cancer stemness but not on EGFR TKI/chemotherapeutic agent sensitivity.

**Fig. 3:**
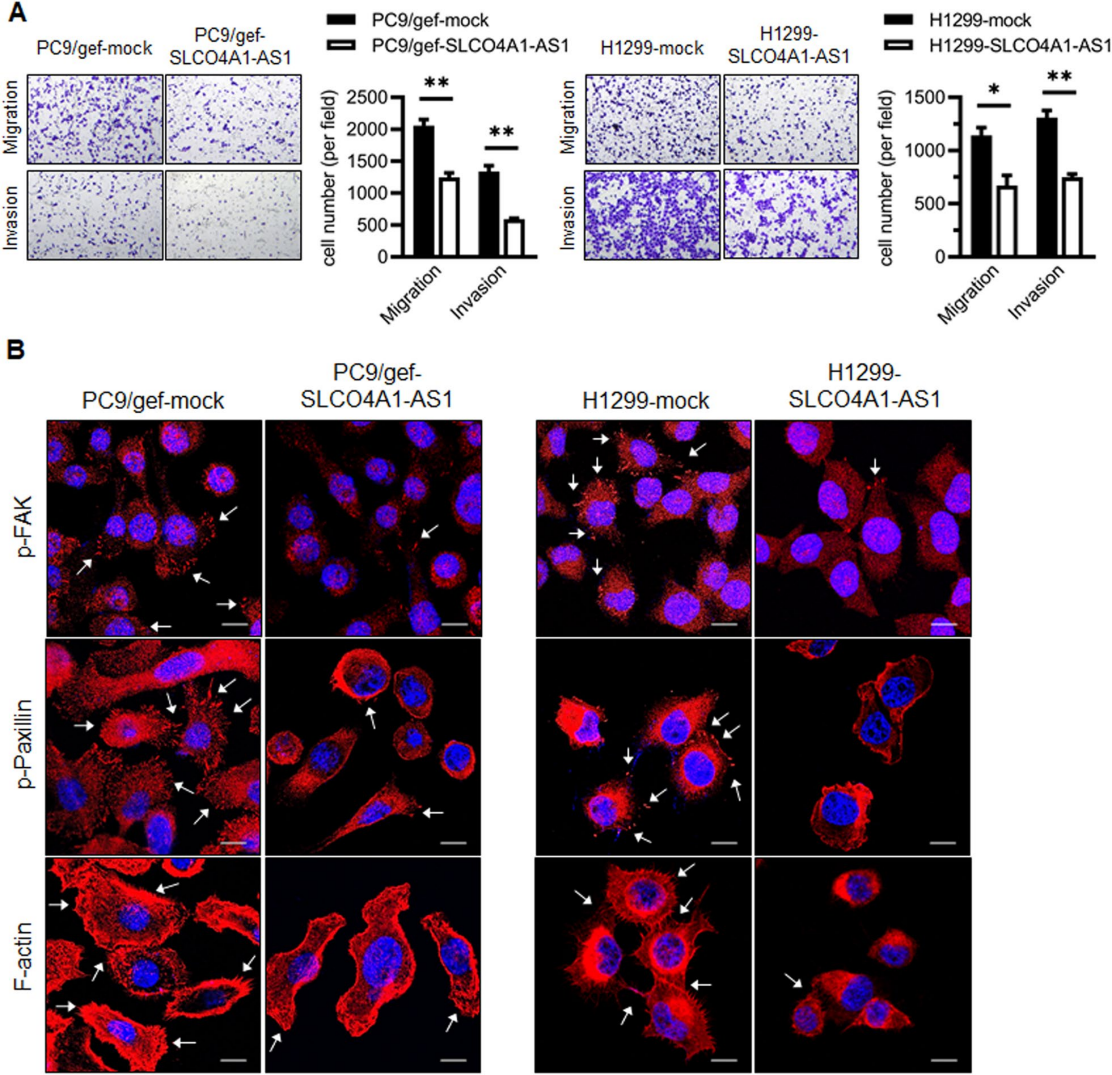
SLCO4A1-AS1 suppresses cell motility.** A** The effect of SLCO4A1-AS1 on migration and invasion of lung cancer cells was performed using migration and invasion assays, respectively. Quantification of migratory and invasive cell numbers are shown (*p < 0.05, **p < 0.01). **B** Immunofluorescence (IF) staining for p-FAK (red), p-paxillin (red), rhodamine-phalloidin for F-actin (red), and nuclei (DAPI, blue) in the control and SLCO4A1-AS1-overexpressing lung cancer cell lines, indicated by white arrows and scale bar = 20 μm

Cell migration and invasion play a significant role in cancer metastasis. Therefore, we evaluated the role of SLCO4A1-AS1 in cell invasion and migration. Overexpression of SLCO4A1-AS1 significantly attenuated cell migration and invasion in the lung cancer cell lines (*p* < 0.05; Fig. 3A; Additional file 8: Fig. S3A). Furthermore, we investigated whether SLCO4A1-AS1 inhibited cell migration and invasion via cytoskeletal remodeling by evaluating the expression of focal adhesion kinase (FAK) and paxillin, which are present beneath the plasma membrane at adhesion sites. Immunofluorescence staining revealed that the fluorescent signals of phospho-FAK and phospho-paxillin around the periphery of cell membranes were decreased in SLCO4A1-AS1 expressing cells (Fig. 3B; Additional file 8: Fig. S3B). Additionally, SLCO4A1-AS1 decreased F-actin (filopodia) formation (Fig. 3B; Additional file 8: Fig. S3B), suggesting that SLCO4A1-AS1 inhibited cell migration and invasion by remodeling the cytoskeleton.

To prevent unforeseeable changes in cancer cell behavior caused by the random genomic integration of SLCO4A1-AS1, we employed transient transfection of SLCO4A1-AS1 to assess its effect on migration and invasion in PC9/gef and H1299 cells. The results showed that transient SLCO4A1-AS1 expression significantly reduced cell migration and invasion in PC9/gef and H1299 cell lines (Additional file 8: Fig. S4A–D).

Furthermore, we utilized siRNA to silence SLCO4A1-AS to assess its impact on the migration and invasion capabilities of PC9/gef and H1299 cells. We found that knockdown of SLCO4A1-AS1 significantly enhanced the migration and invasion ability of PC9/gef and H1299 cells (Additional file 8: Fig. S5A–D). However, knockdown of SLCO4A1-AS1 did not affect *SLCO4A1* mRNA and protein expression (Additional file 8: Fig. S5E, F). Additionally, PC9/gef cell growth was unaffected by SLCO4A1-AS1 knockdown compared with control cells (Additional file 8: Fig. S5G).

Furthermore, an intriguing question arose regarding whether the parental gene of SLCO4A1-AS1, namely *SLCO4A1*, affects cell migration and invasion. To investigate this, we used the siRNA system to knockdown *SLCO4A1* in PC9/gef cells and evaluated its impact on their migration and invasion abilities (Additional file 8: Fig. S6A). Our results indicate that silencing of SLCO4A1 did not affect the migration and invasion abilities, nor did it influence the expression of SLCO4A1-AS1 in *SLCO4A1* knockdown PC9/gef cells (Additional file 8: Fig. S6B, C).

**Fig. 4:**
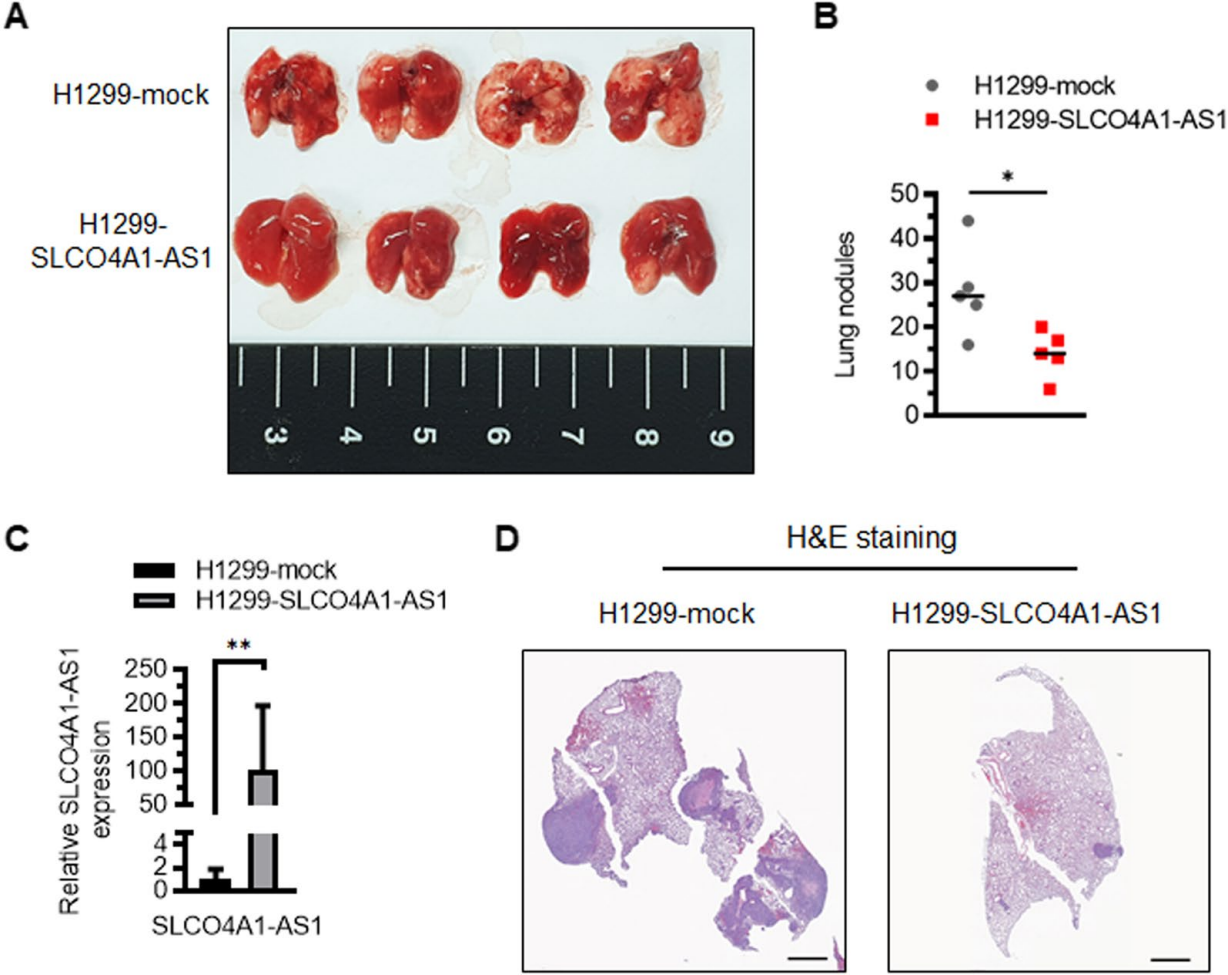
Overexpression of SLCO4A1-AS1 inhibited lung metastasis in NOD/SCID mice.** A** The gross appearance of pulmonary nodules dissected from NOD/SCID mice. **B** The number of lung metastatic nodules were calculated (n = 5 for each group; *p < 0.05). **C** SLCO4A1-AS1 expression from representative lung metastatic nodes. The data of RT-qPCR are presented as mean ± SD (**p < 0.01). **D** Representative H&E staining images of lung tissues sections and scale = 1 mm

We assessed the effect of SLCO4A1-AS1 on tumor metastasis in vivo by injecting SLCO4A1-AS1-overexpressing and control cells (H1299-SLCO4A1-AS1 and H1299-mock) into the tail vein of NOD/SCID mice. After 7 weeks, the mice were sacrificed and the lungs were removed for further examination. We observed that SLCO4A1-AS1-overexpressing cells markedly reduced lung metastatic nodules and pulmonary metastatic lesions (Fig. 4A, B). Histological analyses of the lungs confirmed that overexpression of SLCO4A1-AS1suppressed lung cancer metastasis in vivo (Fig. 4C, D).

Moreover, we evaluated the distant organ metastatic potential between mock-expressing and SLCO4A1-AS1-overexpressing cancer cells. H1299-mock and H1299-SLCO4A1-AS1 cells were injected into mice via tail vein, with 15 mice in each group. After 8–10 weeks, 14 H1299-mock and 11 H1299-SLCO4A1-AS1 cell-injected mice developed lung nodules. Additionally, two H1299-mock cell-injected mice developed malignant ascites as well as liver and kidney metastasis, whereas no liver and kidney metastasis were observed in the H1299-SLCO4A1-AS1 cell-injected mice (Additional file 4: Table S4; Additional file 8: Fig. S7A). These results provide additional evidence that SLCO4A1-AS1 effectively reduces the metastatic ability of lung cancer cells in vivo.

### SLCO4A1-AS1 interacts with TOX4

**Fig. 5:**
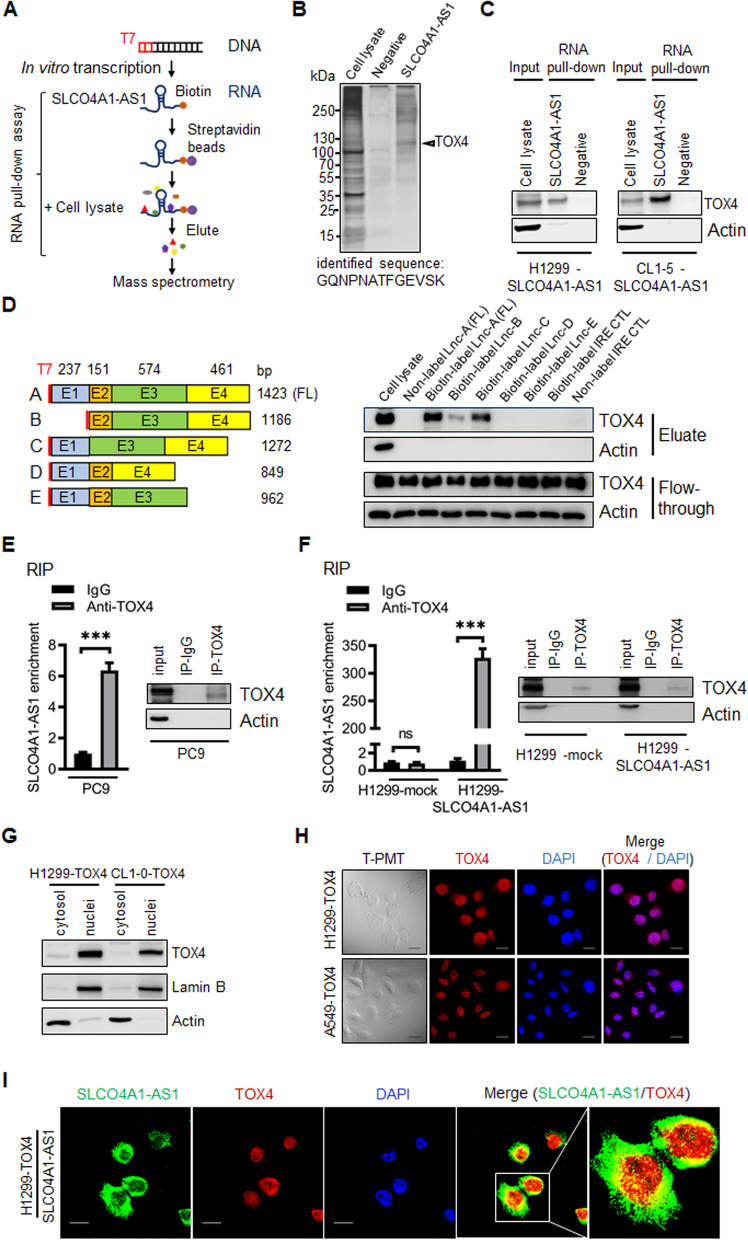
SLCO4A1-AS1 binds to TOX4.** A** Overview of the in vitro RNA pull-down assay and identification of lncRNA SLCO4A1-AS1 associated cellular proteins. **B** Silver staining of biotinylated SLCO4A1-AS1 associated proteins. The SLCO4A1-AS1-specific bands (arrows) were excised and analyzed by mass spectrometry (MS), which were identified as TOX4. **C** Western blotting for proteins from SLCO4A1-AS1 pull-down assays. **D** Western blotting of TOX4 in samples pulled down by full-length (fragment A, including Exon1-4) or truncated SLCO4A1-AS1 (fragment B–D). Exon mapping showed that SLCO4A1-AS1 exon 3 and exon 4 interacted with TOX4 and was indispensable. **E** The PC9 cell lysates were immunoprecipitated with control rabbit IgG or anti-TOX4 antibody, and complexes were analyzed for enrichment of SLCO4A1-AS1 by RT-qPCR. The data are presented as mean ± SD (***p < 0.001). Specific immunoprecipitation of TOX4 was confirmed by western blotting (Inset). **F** TOX4 enriched SLCO4A1-AS1 was confirmed in SLCO4A1-AS1-expressing H1299 cell lysates. RT-qPCR data are presented as mean ± SD (***p < 0.001; ns, not statistically significant). Specific immunoprecipitation of TOX4 was confirmed by western blotting (Inset). **G** Western blot quantification of cytosolic and nuclear protein levels of TOX4 in H1299-TOX4 and CL1-0-TOX4 cells. **H** Immunofluorescence staining showed that TOX4 was localized in the nucleus. Scale bar = 20 μm. **I** RNA fluorescence in situ hybridization (RNA-FISH) and immunofluorescence staining showed that SLCO4A1-AS1 co-localized with TOX4 in SLCO4A1-AS1-expressing H1299-TOX4 cell line. Scale bar = 20 μm

LncRNAs typically exert their functions through physical interactions with various cellular molecules. We performed a protein pull-down assay using biotinylated SLCO4A1-AS1 on three lung cancer cell lines that overexpressed SLCO4A1-AS1 (PC9/gef-SLCO4A1-AS1, CL1-5-SLCO4A1-AS1, and H1299-SLCO4A1-AS1) to identify associated proteins (Fig. 5A). These proteins were analyzed through SDS-PAGE and silver staining (Fig. 5B). Following band screening, we identified 407 candidate target proteins for SLCO4A1-AS1 across the three cell lines using mass spectrometry data intersection (Additional file 5: Table S5). Subsequently, we assessed the target proteins related to cell mobility and found that TOX4 (also known as MIG7) is associated with the invasion ability in glioma cells [32].

The interaction was confirmed by subjecting the pull-down extracts to SDS-PAGE and probing with the TOX4 antibody in H1299-SLCO4A1-AS1 and CL1-5-SLCO4A1-AS1 cells (Fig. 5C). SLCO4A1-AS1 did not affect TOX4 protein expression in SLCO4A1-AS1-overexpressing H1299 or A549 cells (Additional file 8: Fig. S8A). To identify the TOX4-interacting region of SLCO4A1-AS1, we constructed serial plasmids encoding different exon deletion fragments of SLCO4A1-AS1 for in vitro transcription (IVT) to obtain the corresponding transcripts (fragments A–E; Fig. 5D, left). These transcripts were biotinylated and used in pull-down assays. We found that transcripts A, B, and C interacted with TOX4, whereas transcripts D and E inhibited the interaction between SLCO4A1-AS1 and TOX4. This suggests that exons 3 and 4 of SLCO4A1-AS1 are pivotal for their interaction with TOX4 (Fig. 5D, right). To confirm these findings, we performed immunoprecipitation of the TOX4 complex from cell lysates using either an isotype control IgG or an anti-TOX4 antibody and detected the SLCO4A1-AS1 transcript using RT-qPCR. SLCO4A1-AS1 was enriched in anti-TOX4 immunoprecipitation compared with anti-IgG immunoprecipitation (Fig. 5E, F). Furthermore, we assessed the cellular localization of the TOX4 protein using H1299-TOX4 and CL1-0-TOX4 cell fraction lysates and immunofluorescence staining of H1299-TOX4 and A549-TOX4 cells. We found that TOX4 was predominantly localized in the nuclei of lung cancer cells (Fig. 5G, H). RNA-FISH and immunofluorescence confirmed the nuclear co-localization of SLCO4A1-AS1 and TOX4 (Fig. 5I). The co-transfection efficiency was confirmed by RT-qPCR and western blotting (Additional file 8: Fig. S8B, C). Collectively, these results indicated a direct association between TOX4 and SLCO4A1-AS1.

### SLCO4A1-AS1-overexpression reverted TOX4-induced cancer cell migration and invasion

**Fig. 6:**
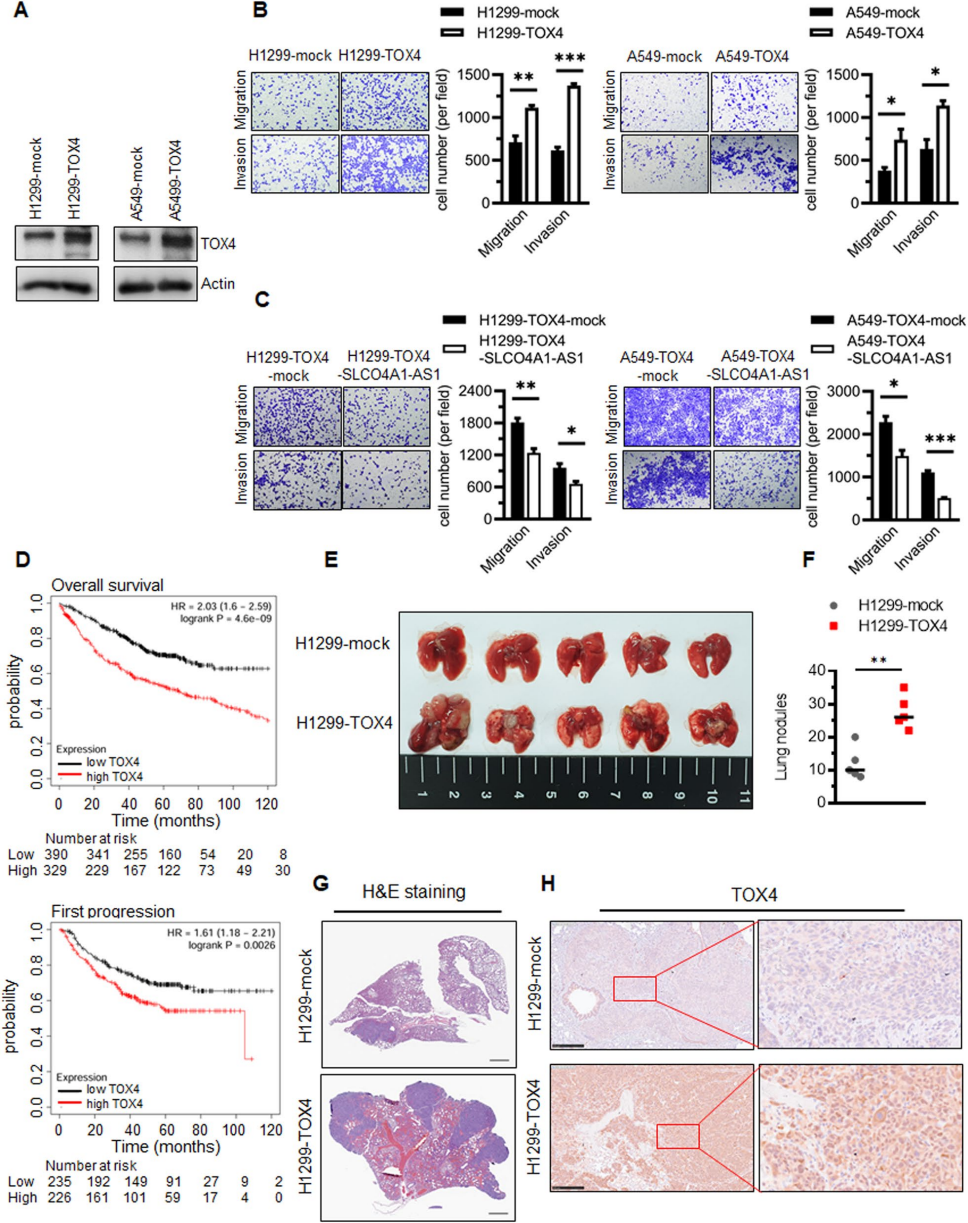
TOX4 facilitates lung cancer cell migration and invasion.** A** Stable overexpression of TOX4 in H1299 and A549 was evaluated by western blotting. **B** The effect of TOX4 overexpression on H1299 and A549 cells migration and invasion was evaluated. Quantification of migratory and invasive cell numbers are shown (*p < 0.05, **p < 0.01, ***p < 0.001). **C** SLCO4A1-AS1 overexpression rescued the TOX4-evoked increase in cells migration and invasion. Quantification of migratory and invasive cell numbers are shown (*p < 0.05, **p < 0.01, ***p < 0.001). **D** The correlation between TOX4 expression and overall survival (log rank test, *p* < 0.001), and first progression (log rank test, *p* = 0.0026) of lung cancer patients from Kaplan Meier plotter database website. **E** The gross appearance of pulmonary nodules dissected from NOD/SCID mice. **F** Number of metastatic nodules in the lung tissue (n = 5 for each group; *p < 0.05). **G** Representative H&E staining images of lung tissues sections and scale = 1 mm. **H** Immunohistochemistry staining to assess TOX4 expression in metastatic lung node. Scale = 1 mm and 250 μm

We investigated whether TOX4 modulates cell migration and invasion by overexpressing TOX4 in H1299 and A549 cells (Fig. 6A) and silencing TOX4 in H1299 and CL1-5 cells (Additional file 8: Fig. S9A). TOX4 knockdown significantly reduced cell migration and invasion in both H1299 and CL1-5 cells (Additional file 8: Fig. S9B). In contrast, TOX4-overexpression augmented lung cancer cell migration and invasion (Fig. 6B). Phospho-FAK, phospho-paxillin, and F-actin levels increased in TOX4-overexpressing H1299 cells (Additional file 8: Fig. S10A). Furthermore, the overexpression of SLCO4A1-AS1 counteracted the TOX4-induced elevation of phospho-FAK, phospho-paxillin, and F-actin levels, thereby affecting the cytoskeletal structure (Additional file 8: Fig. S11A) and mitigating TOX4-induced migration and invasion (Fig. 6C). Moreover, survival analysis using the Kaplan–Meier plotter revealed that patients with lung adenocarcinoma who had higher TOX4-expressing tumors had shorter overall survival and recurrence than patients with lower TOX4-expressing tumors (Fig. 6D).

In the mouse tail vein metastasis model, TOX4-overexpression significantly increased the formation of metastatic nodules in the lungs (Fig. 6E, F). Histological analysis of the lungs confirmed lung metastases in different groups (Fig. 6G). Immunohistochemistry of the resected lungs validated the role of TOX4 in the promotion of lung cancer metastasis in vivo (Fig. 6H). These results suggest that TOX4 is essential for lung cancer cell migration and invasion, and is crucial for the promotion of lung cancer progression.

### SLCO4A-AS1 interrupted the binding of TOX4 to *NTSR1* promoter and suppressed the expression of NTSR1

**Fig. 7:**
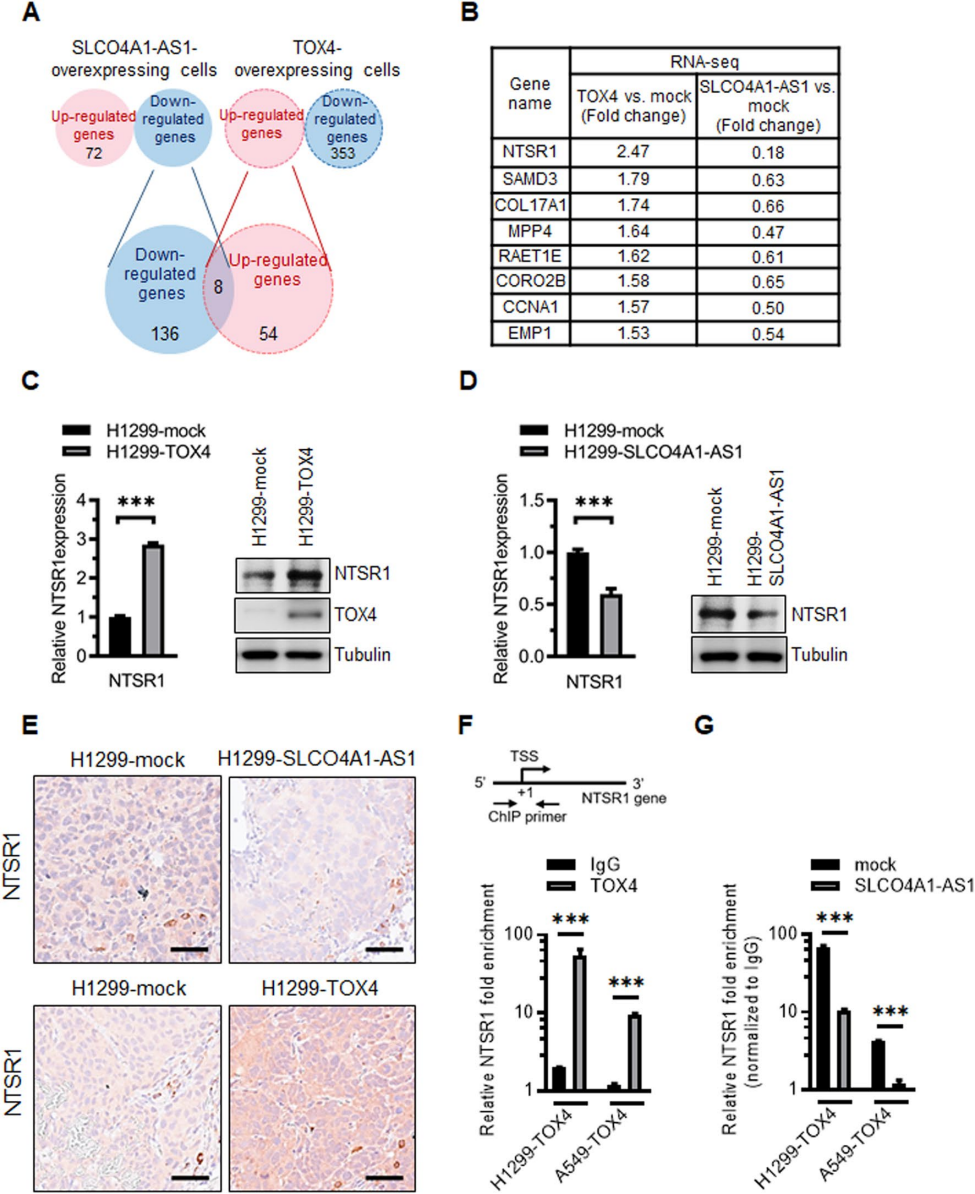
Identification of downstream target for the SLCO4A1-AS1/TOX4 axis.** A** Screening of candidate genes using intersection of two pairs of RNA-seq data (H1299-mock vs. H1299-TOX4 and H1299-mock vs. H1299-SLCO4A1-AS1). The eight protein coding candidate genes were upregulated in TOX4-overexpressing cells and downregulated in SLCO4A1-AS1-overexpressing cells. **B** The fold change of candidate genes was validated in the TOX4-overexpressing cells and SLCO4A1-AS1-overexpressing H1299 cells by RT-qPCR assay. **C***NTSR1* mRNA and protein expression was detected in TOX4-expressing H1299 cells by RT-qPCR and western blotting. RT-qPCR data are presented as mean ± SD (***p < 0.001). **D***NTSR1* mRNA and protein expression was detected in SLCO4A1-AS1-expressing H1299 cells by RT-qPCR and western blotting. RT-qPCR data are presented as mean ± SD (***p < 0.001). **E** Immunohistochemistry staining was performed to evaluated NTSR1 expression in mouse tail vein lung metastasis model and scale = 50 μm. **F** Chromatin immunoprecipitation (ChIP) was performed with an antibody against TOX4 or IgG in H1299-TOX4 and A549-TOX4 cells. The enriched *NTSR1* promoter was purified and quantified by RT-qPCR using primers targeting the *NTSR1* promoter region (inset). The data of RT-qPCR are presented as mean ± SD (***p < 0.001). **G** ChIP was performed to assess the effect of SLCO4A1-AS1 overexpression on the interaction of TOX4 and *NTSR1* promoter. The *NTSR1* promoter was quantified by RT-qPCR using primers targeting the *NTSR1* promoter region. The data of RT-qPCR are presented as mean ± SD (***p < 0.001)

We explored the molecular mechanisms governed by SLCO4A1-AS1 and TOX4 by analyzing differential gene expression between two paired cell lines (H1299-mock vs. H1299-SLCO4A1-AS1 and H1299-mock vs. H1299-TOX4) using RNA-seq. We focused on protein-coding genes selected from the RNA-seq data and found that 54 coding genes were upregulated and 353 were downregulated in H1299-TOX4 cells (*p* < 0.05; fold change > 1.5). Additionally, 72 coding genes were upregulated and 136 were downregulated in H1299-SLCO4A1-AS1 cells (Fig. 7A). Furthermore, eight candidate target proteins were simultaneously regulated in TOX4- and SLCO4A1-AS-overexpressing cells (Fig. 7A, B; Additional file 6: Table S6 and Additional file 7: Table S7). Notably, NTSR1 (neurotensin receptor 1) was the most promising target within the SLCO4A1-AS1/TOX4 axis. NTSR1 was upregulated by 2.5-fold in TOX4-overexpressing cells and downregulated 0.18-fold in SLCO4A1-AS1-overexpressing cells compared to the corresponding control cells (Fig. 7B). Further validation revealed that TOX4-overexpression significantly increased *NTSR1* mRNA and protein expression in H1299 and A549 cells (Fig. 7C; Additional file 8: Fig. S12A), whereas NTSR1 expression significantly decreased in SLCO4A1-AS1-expressing cells (Fig. 7D; Additional file 8: Fig. S12B). To clarify whether different transcripts of SLCO4A1-AS1 exhibited differential effects on the downstream NTSR1, we established three different SLCO4A1-AS1 transcript-expressing PC9/gef cells. Among them, the full-length transcript SLCO4A1-AS1:4, encompassing all four exons, exhibited a more pronounced suppression of NTSR1. In contrast, transcriptsSLCO4A1-AS1:1 and 1:2, lacking exon 2 and 3 for TOX4 recruitment, showed diminished regulatory capacity (Additional file 8: Fig. S12C, D). Consistent with the data from cultured cells, NTSR1 was downregulated in SLCO4A1-AS1 overexpressing lung tumors and upregulated in TOX4-overexpressing lung tumor tissues from a mouse tail vein metastasis model (Fig. 7E). Given that TOX4 is a DNA-binding protein, we investigated whether TOX4 is a potential transcriptional regulator of *NTSR1*. First, we determined whether TOX4 binds to the *NTSR1* promoter through ChIP assay in H1299-TOX4 and A549-TOX4 cells. The region spanning from − 10 to + 90 relative to the transcription start site (TTS, + 1) of the *NTSR1* promoter exhibited significant enrichment upon TOX4 immunoprecipitation (Fig. 7F). These results indicated that TOX4 binds to the *NTSR1* promoter and regulates NTSR1 protein expression. Furthermore, overexpression of SLCO4A1-AS1 interrupted the interaction between TOX4 and the *NTSR1* promoter (Fig. 7G), indicating that TOX4 is a transcriptional regulator of *NTSR1* and that the binding of TOX4 to the *NTSR1* promoter can be blocked by SLCO4A1-AS1.

We also conducted *NTSR1* promoter luciferase assays to determine whether TOX4 activated the transcription of *NTSR1*. We used cell pairs with differential expression levels of TOX4 (H1299-TOX4 and H1299-mock; PC9 and PC9/gef). We observed a higher expression level of endogenous TOX4 and *NTSR1* promoter luciferase activity in PC9/gef cells compared to PC9 cells (Additional file 8: Fig. S12E). Furthermore, overexpression of TOX4 in H1299 cells resulted in a significant increase in luciferase activity in TOX4-overexpressing H1299 compared to corresponding control H1299-mock cells (Additional file 8: Fig. S12F). Additionally, overexpression of SLCO4A1-AS1 reversed TOX4-promoted transcriptional activity of the *NTSR1* promoter in PC9/gef cells (Additional file 8: Fig. S12G). Therefore, we suggest that TOX4 may activate the transcription of *NTSR1*.

### NTSR1 is essential in SLCO4A1-AS1/TOX4-mediated migration and invasion

**Fig. 8:**
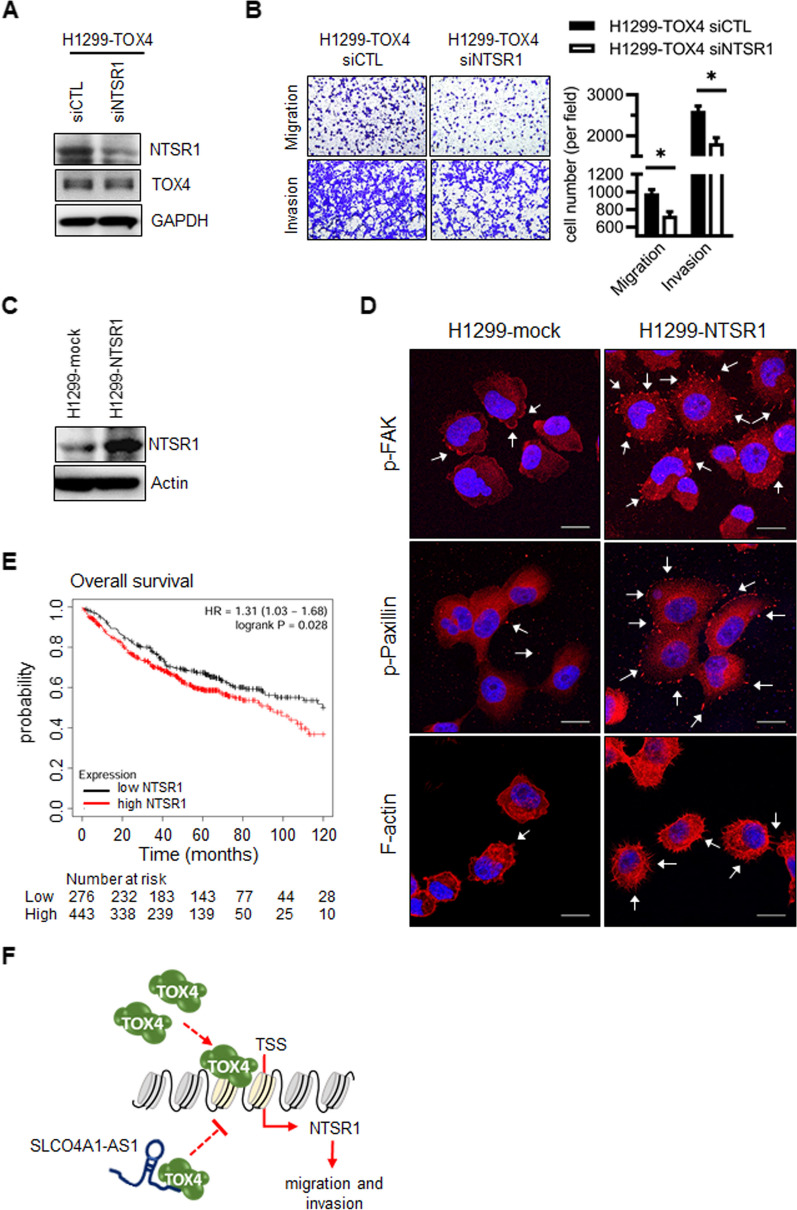
SLCO4A1-AS1/TOX4-mediate migration and invasion through NTSR1.** A** H1299-TOX4 cells were transfected with NTSR1 small interfering RNAs (siNTSR1) or scramble siRNA (siCTL). The effect of siRNAs was evaluated by western blotting. **B** The effect of NTSR1 knockdown on H1299-TOX4 cell migration and invasion was measured. Quantification of migratory and invasive cell numbers are shown (*p < 0.05). **C** Stable overexpression of NTSR1 in H1299 was evaluated by western blotting. **D** IF staining for p-FAK (red), p-paxillin (red), rhodamine-phalloidin for F-actin (red), and nuclei (DAPI, blue) in the control and H1299-NTSR1 cells, indicated by white arrows and scale bar = 20 μm. **E** The correlation between NTSR1 expression and overall survival of lung adenocarcinoma patients from Kaplan Meier plotter database website (log rank test, *p* = 0.028). **F** Hypothetical model for the tumor suppressor roles of SLCO4A1-AS1 in lung cancer

We verified the role of NTSR1 in TOX4-mediated cell movement by silencing NTSR1 in H1299-TOX4 and A549-TOX4 cells and examined their migratory and invasive abilities. The loss-of-function assay showed that *NTSR1* knockdown significantly reduced the migration and invasion of H1299-TOX4 and A549-TOX4 cells (Fig. 8A, B; Additional file 8: Fig. S12H, I). Conversely, NTSR1-overexpression in H1299 and A549 cells increased phosphorylation of FAK and paxillin, and remodeled the F-actin structure (Fig. 8C and D; Additional file 8: Fig. S12J, K). According to the Kaplan–Meier plotter database, higher NTSR1 expression was associated with shorter overall survival in patients with lung adenocarcinoma (Fig. 8E). The evidence showed that *NTSR1* is a novel and convergent downstream target of SLCO4A1-AS1/TOX4 and is involved in regulating SLCO4A1-AS1/TOX4-mediated migration and invasion in lung cancer cells. In summary, SLCO4A1-AS1 exerts its inhibitory influence on NTSR1 expression through its interaction with TOX4, thereby exerting a modulatory control over the migratory and invasive behaviors of lung cancer cells (Fig. 8F).

## Discussion

Li et al. [22] revealed elevated expression of the SLCO4A1-AS1 transcript in tumor tissues compared with adjacent non-tumor tissues in a Chinese patient cohort. They established that SLCO4A1-AS1 functions as a competing endogenous non-coding RNA, sequestering miR-223-3p to enhance IKKα expression, which in turn activates the IKK/NF-κB pathway. Using the shRNA system to suppress SLCO4A1-AS1 resulted in the inhibition of the IKK/NF-κB pathway, which led to reduced migration, invasion, and proliferation in lung cancer cells A549 and H1299. However, we found that SLCO4A1-AS1-overexpression reduced cell migration and invasion, whereas its suppression promoted cell migration and invasion in PC9/gef and H1299 cell lines (Additional file 8: Fig. S5A–D). Rigorous cell line authentication, verified through short tandem repeat (STR) analysis, corroborated the identity of the cell lines with supplier specifications (Additional file 8: Fig. S17A–F). Furthermore, we used two independent cohorts to analyze the expression of the SLCO4A1-AS1 transcript in lung adenocarcinomas, including The Cancer Genome Atlas (TCGA) dataset and Taiwanese patient cohort [29]. In these cohorts, the expression of SLCO4A1-AS1 was lower in tumor tissues than in adjacent non-tumor tissues (Fig. 1C, D). The association between SLCO4A1-AS1 and survival across several Gene Expression Omnibus datasets, including (GSE31210), revealed a positive correlation between elevated SLCO4A1-AS1 expression and longer overall survival and progression-free survival (Fig. 1E). These results support the notion that SLCO4A1-AS1 exerts a tumor-suppressive role.

To our knowledge, this is the first study to demonstrate that SLCO4A1-AS1 expression is significantly reduced in highly metastatic lung cancer cells. Furthermore, we have identified the physiological functions of SLCO4A1-AS1 and determined its molecular mechanism of action. We determined that SLCO4A1-AS1 is downregulated in lung cancer tissue, and its ectopic expression inhibits the migration and invasion capabilities of lung cancer cells. Furthermore, SLCO4A1-AS1 decreased phosphorylated-FAK and phosphorylated-paxillin, disrupted cytoskeleton filaments, and reduced cell motility. Moreover, we used mass spectrometry, RNA pull-down, and RNA-IP to demonstrate that SLCO4A1-AS1 interacts with TOX4 and inhibits TOX4-dependent migration and invasion. Additionally, we identified *NTSR1* as the downstream target of TOX4 and found that SLCO4A1-AS1 acts as an antagonist of TOX4 to reduce the transcription of *NTSR1*. Furthermore, SLCO4A1-AS1 antagonizes NTSR1-mediated cytoskeletal remodeling to inhibit migration and invasion. Our comprehensive study underscores the tumor-suppressive role of SLCO4A1-AS1, orchestrating the intricate regulatory mechanisms underlying the migration, invasion, and metastasis of lung cancer cells.

Various splicing transcripts of lncRNAs exhibit different characteristics [33]. The interaction between lncRNAs and target proteins depends on the SLCO4A1-AS1 transcript sequences. Compared to the SLCO4A1-AS1:4 transcript, both SLCO4A1-AS1:1 and SLCO4A1-AS1:2 spliced transcripts were deficient in exons 2 and 3. Exons 3 and 4 of SLCO4A1-AS1 are essential for recruiting TOX4, suggesting that the spliced transcripts of SLCO4A1-AS1:1 and SLCO4A1-AS1:2 may not antagonize TOX4-depnedent migration and invasion. Owing to the variety of lncRNA transcripts, they efficiently modulate a wide range of effects in cancer. In this study, we demonstrated that SLCO4A1-AS1 participates in cancer cell migration and invasion by physically sequestering TOX4 and downregulating TOX4-dependent transcription.

TOX4, a member of the high-mobility group (HMG)-box superfamily, contains a DNA-binding domain that is highly conserved across species. However, its function is poorly understood. The currently known function of TOX4 is to regulate chromatin structure during mitosis to interphase transition, influence cell cycle and cell death through TOX4-containing complexes (such as PNUTS and PP1), and participate in transcription initiation and elongation of downstream targets [34, 35]. Additionally, TOX4 is involved in the expression of stemness-related markers (Oct4, Klf4, Sox2, and Myc) [35]. A recent study showed that TOX4 regulates transcription by promoting the dephosphorylation of the RNA polymerase II C-terminal domain [36]. These findings suggest an important role for TOX4 in controlling gene expression, cell survival, and cancer progression. In our study, we found that TOX4 knockdown severely reduced the migration and invasion of lung cancer cells; however, TOX4-overexpression enhanced cell migration and invasion. Therefore, we speculate that TOX4 promotes cancer cell migration and invasion by regulating downstream genes involved in cell motility.

NTSR1 is a G-protein coupled receptor (GPCR) that mediates several biological processes through its interaction with neurotensin (NTS) peptide [37, 38]. Previous studies have demonstrated that the NTS/NTSR1 complex plays an essential role in regulating tumorigenesis, proliferation, apoptosis, metastasis, and differentiation in a variety of cancers [39–41], primarily through pathways such as Rho GTPases/FAK, IP3/DAG/Ca2^+^ release, and PKC/RAF-1/MAPKs [38]. Higher expression of NTS and NTSR1 has been observed in lung cancer tissues than in normal tissues [42]. NTSR1 expression is strongly associated with poor prognosis [41, 43]. Previous studies support our finding that NTSR1-overexpression enhances lung cancer cell migration and invasion, increases FAK and paxillin phosphorylation, and affects F-actin distribution. In this study, we have discovered that TOX4 directly binds to the *NTSR1* promoter and enhances transcription of the *NTSR1* gene in lung cancer cells.

Metastasis is the leading cause of cancer-related deaths. A comprehensive understanding of the molecular mechanisms underlying tumor cell migration and metastasis is crucial for the development of novel therapeutic interventions for aggressive and metastatic conditions. In this study, we demonstrated that SLCO4A1-AS1 acts as an antagonist that reduces the availability of TOX4 to the *NTSR1* promoter. SLCO4A1-AS1 reverses TOX4/NTSR1-dependent migration and invasion and reduces lung cancer cell motility by remodeling the cytoskeleton. The regulatory network of SLCO4A1-AS1, TOX4, and NTSR1 in regulating cancer migration and invasion may provide potential anti-metastatic therapeutic targets for the treatment of lung cancer.

## Conclusion

This study underscores the pivotal role of SLCO4A1-AS1, which functions as a decoy to sequester TOX4, attenuating its availability for transcriptional activity. Consequently, this leads to a reduction in NTSR1 expression, ultimately inhibiting lung cancer migration and invasion. This study unravels the intricate SLCO4A1-AS1/TOX4/NTSR1 axis and may provide a potential avenue for therapeutic intervention for NSCLC.

### Supplementary Information

**Additional file 1: Table S1.** List of primers for quantitative real-time PCR.

**Additional file 2: Table S2.** Antibodies for immunocytochemistry, western blotting, and immunoprecipitation assays.

**Additional file 3: Table S3.** LncRNAs showing differential expression in PC9/gef versus PC9 lung cancer cells.

**Additional file 4: Table S4.** The proportion of metastatic sites generated in mice with lung tumors.

**Additional file 5: Table S5.** Intersection data from mass spectrometry was used to identify candidate target proteins of SLCO4A1-AS1 in three overexpressing cell lines (PC9/gef-SLCO4A1-AS1, CL1-5-SLCO4A1-AS1, and H1299-SLCO4A1-AS1).

**Additional file 6: Table S6.** Protein coding genes showing differential expression in H1299-mock versus H1299-SLCO4A1-AS1 lung cancer cells.

**Additional file 7: Table S7.** Protein coding genes showing differential expression in H1299-mock versus H1299-TOX4 lung cancer cells.

**Additional file 8: Figure S1.** SLCO4A1-AS1 overexpression inhibits cancer stem cells properties. **A** Stable overexpression of SLCO4A1-AS1 was confirmed by RT-qPCR. The data of RT-qPCR are presented as mean ± SD (***p < 0.001). **B** Morphology and **C** average number of spheres per well of control and SLCO4A1-AS1-overexpressing lung cancer cells were calculated in the sphere formation assay (**p < 0.01, ***p < 0.001). Scale bar = 50 µm. **D** Overexpression of SLCO4A1-AS1 decreased the proportion of ALDH^+^ lung cancer cells using the Aldefluor™ assay (**p < 0.01, ***p < 0.001). DEAB is an ALDH inhibitor used to improve assay specificity. **E** RT-qPCR was performed to measure the expression levels of ALDH1A1 in SLCO4A1-AS1-overexpressing lung cancer cells. RT-qPCR data are presented as mean ± SD (*p < 0.05, **p < 0.01, ***p < 0.001). **F** The SLCO4A1-AS1-overexpressing cells and control cells were stained with Hoechst 33342 and then the side population is gated and distinguished by the treatment of verapamil (vera). The results from four independent experiments were quantified on the right, with statistical significance (***p < 0.001) observed. **Figure S2.** The effects of overexpressing SLCO4A1-AS1 on cell proliferation and drug resistance. **A** Cell growth was assessed using the CCK-8 assay in SLCO4A1-AS1 expression PC9/gef and H1299 cell lines (ns, not statistically significant). **B** CCK-8 assay for SLCO4A1-AS1 expressing PC9/gef and H1299 cells treated with increasing concentrations of osimertinib and paclitaxel for 96 hours, respectively (ns, not statistically significant).**Figure S3.** SLCO4A1-AS1 suppresses cell motility. **A** The effect of SLCO4A1-AS1-overexpressing CL1-5 cells on migration and invasion was performed using transwell and matrigel assays, respectively. Quantification of migratory and invasive cell numbers are shown (*p < 0.05; ns, not statistically significant). **B** Immunofluorescence staining for p-FAK (red), p-paxillin, (red), rhodamine-phalloidin for F-actin (red), and nuclei (DAPI, blue) in the control and SLCO4A1-AS1-overexpressing CL1-5 cells, indicated by white arrows and scale bar = 20 µm. **Figure S4.** The transient expression of SLCO4A1-AS1 inhibits lung cancer cells migration and invasion. Transient expression of SLCO4A1-AS1 was confirmed in **A** PC9/gef and **C** H1299 cells. These data of RT-qPCR are presented as mean ± SD (***p < 0.001). The effect of SLCO4A1-AS1 transient expression on migration and invasion of **B** PC9/gef and **D** H1299 cells was evaluated. Quantification of migratory and invasive cell numbers are shown (*p < 0.05, **p < 0.01). **Figure S5.** Knockdown of SLCO4A1-AS1 promotes lung cancer cell migration and invasion. The efficiency of knockdown SLCO4A1-AS1 in **A** PC9/gef cells and **B** H1299 through siRNA transfection was confirmed by RT-qPCR. The data of RT-qPCR are presented as mean ± SD (***p < 0.001). The effect of SLCO4A1-AS1 knockdown on migration and invasion of **C** PC9/gef and **D** H1299 cells was measured using transwell and matrigel assays, respectively. Quantification of migratory and invasive cell numbers are shown (*p < 0.05, **p < 0.01, ***p < 0.001). **E** The effect of SLCO4A1-AS1 knockdown on *SLCO4A1* mRNA expression was evaluated by RT-qPCR (ns, not statistically significant). **F** The impact of SLCO4A1-AS1 knockdown on SLCO4A1 protein levels was assessed by western blotting assay. **G** Cell growth in SLCO4A1-AS1-silenced PC9/gef cells was assessed using the CCK-8 assay (ns, not statistically significant). **Figure S6.** Knockdown of SLCO4A1 did not affect the migration and invasion abilities of PC9/gef cells. **A** The efficiency of knockdown SLCO4A1 in PC9/gef cells through siRNA transfection was confirmed by RT-qPCR and western blotting. The data of RT-qPCR are presented as mean ± SD (**p < 0.01). **B** The effect of SLCO4A1 knockdown on migration and invasion of PC9/gef cells was measured using transwell and matrigel assays, respectively. Quantification of migratory and invasive cell numbers are shown (ns, not statistically significant). **C** The effect of SLCO4A1 knockdown on lncRNA SLCO4A1-AS1 expression was evaluated by RT-qPCR (ns, not statistically significant). **Figure S7.** SLCO4A1-AS1 inhibits metastases in vivo. **A** Representative H&E staining images of lung, kidney, and liver tissue sections from NOD/SCID mice injected with H1299-mock (n = 15) or H1299-SLCO4A1-AS1 (n = 15), with a scale of 500 µm. **Figure S8.** SLCO4A1-AS1 did not affect the TOX4 expression levels. **A** TOX4 protein expression was detected in SLCO4A1-AS1-expressing H1299 and A549 cells by western blotting. **B** SLCO4A1-AS1 overexpression in H1299-TOX4 and A549-TOX4 cells was evaluated by RT-qPCR assay. The data of RT-qPCR are presented as mean ± SD (***p < 0.001). **C** The TOX4 protein levels in SLCO4A1-AS1-expressing H1299-TOX4 and A549-TOX4 cells were measured by western blotting. **Figure S9.** TOX4 knockdown inhibits lung cancer cell migration and invasion. **A** H1299 and CL1-5 cells were transfected with TOX4 small interfering RNAs (siTOX4) or scramble siRNA (siCTL). The effect of siRNAs was evaluated by western blotting. **B** The effect of TOX4 knockdown on H1299 and CL1-5 cell migration and invasion were measured using transwell and matrigel assays, respectively. Quantification of migratory and invasive cell numbers are shown (*p < 0.05, **p < 0.01, ***p < 0.001). **Figure S10.** TOX4 promotes cell motility. **A** Immunofluorescence staining for p-FAK (red), p-paxillin, (red), rhodamine-phalloidin for F-actin (red), and nuclei (DAPI, blue) in the control and TOX4-overexpressing H1299 cells, indicated by white arrows and scale bar = 20 µm. **Figure S11.** SLCO4A1-AS1 rescues the TOX4-promoted cell motility. **A** Immunofluorescence staining for p-FAK (red), p-paxillin, (red), rhodamine-phalloidin for F-actin (red), and nuclei (DAPI, blue) in the control and SLCO4A1-AS1-expressing H1299-TOX4 and A549-TOX4 cells, indicated by white arrows and scale bar = 20 µm. **Figure S12.** *NTSR1* is the SLCO4A1-AS1/TOX4 downstream target. **A** NTSR1 protein expression was detected in TOX4-expressing A549 cells by western blotting. **B** The protein expression of NTSR1 was detected in SLCO4A1-AS1-expressing A549 cells using western blotting. **C** The expression level of SLCO4A1-AS1 transcripts were evaluated with transcript specific primers by RT-qPCR assay. **D** The protein expression level of NTSR1 were determined in different SLCO4A1-AS1 transcript-expressing PC9/gef cells. **E** Western blotting was used to detect TOX4 and *NTSR1* promoter luciferase assay was performed in PC9 and PC9/gef cells after transfection of pGL3/NTSR1 luciferase vector or pGL3 empty vector. **F** *NTSR1* promoter luciferase assay was done by transfecting H1299-mock and H1299-TOX4 cells with pGL3/NTSR1 luciferase vector or pGL3 empty vector. **G** *NTSR1* promoter luciferase assay was done by transfecting PC9/gef-mock and PC9/gef-SLCO4A1-AS1 cells with pGL3/NTSR1 luciferase vector or pGL3 empty vector. All the relative luciferase activities are presented as means ± SD (***p < 0.001). **H** A549-TOX4 cells were transfected with NTSR1 small interfering RNAs (siNTSR1) or scramble siRNA (siCTL). The effect of siRNAs was evaluated by western blotting.**I** The effect of NTSR1 knockdown on A549-TOX4 cell migration and invasion were measured using transwell and matrigel assays, respectively. Quantification of migratory and invasive cell numbers are shown (*p < 0.05). **J** Stable overexpression of NTSR1 in A549 was evaluated by western blotting. **K** Immunofluorescence staining for p-FAK (red), p-paxillin, (red), rhodamine-phalloidin for F-actin (red), and nuclei (DAPI, blue) in the control and A549-NTSR1 cells, indicated by white arrows and scale bar = 20 µm. **Figure S13.** Original films refer to Fig. 5B–G. **Figure S14.** Original films refer to Fig. 6A. **Figure S15.** Original films refer to Fig. 7C, D. **Figure S16.** Original films refer to Fig. 8A, C. **Figure S17.** STR DNA profile of lung cancer cell lines. **A** PC9, **B** PC9/gef, **C** H1299, **D** A549, **E** CL1-5, and **F** CL1-0.

**Additional file 9.** Additional methods.

## Data Availability

The original films for each western blot and STR DNA profile of lung cancer lines are shown in Additional file 8: Fig. S13–S17. Supplementary methods are attached in Additional file 9. Expression profile data were obtained from GEO at GSE60189. Additional gene expression profiles are available in Additional files. Mass spectrometry proteomics data and materials used in the study are available from the corresponding author upon reasonable request.

## Abbreviations

ceRNA: Competing endogenous RNA
ChIp: Chromatin immunoprecipitation
DEG: Differentially expressed gene
EMT: Epithelial–mesenchymal transition
FISH: Fluorescence in situ hybridization
GEO: Gene expression omnibus
GO: Gene ontology
HBSS: Hanks’ balanced salt solution
IVT: In vitro transcription
LncRNA: Long non-coding RNA
NSCLC: Non-small cell lung cancer
NTSR1: Neurotensin receptor 1
PRC2: Polycomb repressive complex 2
RIP: RNA immunoprecipitation
RNA-seq: RNA sequencing
RT-qPCR: Reverse transcription quantitative polymerase chain reaction
siRNAs: Small interfering RNAs
SLCO4A1-AS1: Solute carrier organic anion transporter family member 4A1-antisense 1
TBP: TATA box-binding protein
TSS: Transcription start site
