# Supplementary material for: LncRNA SLCO4A1-AS1 suppresses lung cancer progression by sequestering the TOX4-NTSR1 signaling axis
Source: J Biomed Sci. 2023 Sep 19;30:80. doi: 10.1186/s12929-023-00973-9 (PMC10507979; doi:10.1186/s12929-023-00973-9)
Supplement: Supplementary file 1 — Additional file 1: Table S1. List of primers for quantitative real-time PCR. [file 12929_2023_973_MOESM1_ESM.pdf]

Additional Table

Table S1. List of primers for quantitative real-time PCR

Gene name	Primer
**RT-qPCR**	
SLCO4A1-AS1:1-F	5'-GGCACCACCAGTGTC GCT-3'
SLCO4A1-AS1:1-R	5'-TGAGAGGCTGGAAAGTGTAGGTTT A-3'
SLCO4A1-AS1:2-F	5'-CACCACCAGCCTCTCACCA-3'
SLCO4A1-AS1:2-R	5'-GTTCCAGGCATTCAGAGTTGC-3'
SLCO4A1-AS1:4-F	5'-GCAGAGTGTCGCTGACTTGAA-3'
SLCO4A1-AS1:4-R	5'-TGAGAGGCTGGAAAGTGTAGGTT-3'
SLCO4A1-F	5'-GCCATGAGCCGCTACATACTC-3'
SLCO4A1-R	5'-CAGACAAGTTTCCAGGCCATCT-3'
NTSR1-F	5'-TGGACTCCGTTCCTCTATGAC-3'
NTSR1-R	5'-AAGTTGGCAGAGACGAGGTT-3'
ALDH1A1-F	5'-CCAAAGACATTGATAAAGCCATAA-3'
ALDH1A1-R	5'-CACGCCATAGCAATTCACC-3'
TBP-F	5'-CACGAACCACGGCACTGATT-3'
TBP-R	5'-TTTTCTTGCTGCCAGTCTGGAC-3'
**ChIP**	
ChIP-NTSR1-F	5'-TCCCGGCGCCACAAG-3'
ChIP-NTSR1-R	5'-TCCAGGTGCCTCCGATCT-3'
