# Supplementary material for: LncRNA SLCO4A1-AS1 suppresses lung cancer progression by sequestering the TOX4-NTSR1 signaling axis
Source: J Biomed Sci. 2023 Sep 19;30:80. doi: 10.1186/s12929-023-00973-9 (PMC10507979; doi:10.1186/s12929-023-00973-9)
Supplement: Supplementary file 2 — Additional file 2: Table S2. Antibodies for immunocytochemistry, western blotting, and immunoprecipitation assays. [file 12929_2023_973_MOESM2_ESM.pdf]

Additional Table

Table S2. Antibodies for immunocytochemistry, western blotting, and immunoprecipitation assays

Name	Catalog number	Company	Application
rhodamine phalloidin	#R415	Invitrogen	IF
phospho-FAK (Y397)	#44-625G	Invitrogen	IF
phospho-paxillin (Y31)	#ab32115	Abcam	IF
Alexa Fluor™ 555 Phalloidin	#A34055	Invitrogen	IF
Goat anti-Rabbit IgG (H+L) Cross-Adsorbed Secondary Antibody, Alexa Fluor™ 555	# A-21428	Invitrogen	IF
SLCO4A1	#ARP85243_P050	AVIVA	WB
TOX4	#HPA027551	Sigma-Aldrich	IF, IHC
DAPI	#62248	Thermo Fisher Scientific	IF
TOX4	#A304-873A-T	Bethyl	WB, IP
NTSR1	#Ab117592	Abcam	WB, IHC
Lamin B	#sc-6210	Santa Cruz	WB
GAPDH	#5174	Cell Signaling Technology	WB
β-actin	#MAB1501	Millipore	WB
α-tubulin	#Gex102078	Genetex	WB

IF: immunofluorescence; IHC: immunohistochemistry; IP: immunoprecipitation; WB: Western blotting.
