# Supplementary material for: LncRNA SLCO4A1-AS1 suppresses lung cancer progression by sequestering the TOX4-NTSR1 signaling axis
Source: J Biomed Sci. 2023 Sep 19;30:80. doi: 10.1186/s12929-023-00973-9 (PMC10507979; doi:10.1186/s12929-023-00973-9)
Supplement: Supplementary file 4 — Additional file 4: Table S4. The proportion of metastatic sites generated in mice with lung tumors. [file 12929_2023_973_MOESM4_ESM.pdf]

Additional Table

Table S4. The proportion of metastatic sites generated in mice with lung tumors

The cells of tail vein injection	Brain	Liver	Kidney	Muscle
**H1299-mock**	0/14	2/14	2/14	8/14
**H1299-SLCO4A1-AS1**	0/11	0/11	0/11	6/11
