# Supplementary material for: LncRNA SLCO4A1-AS1 suppresses lung cancer progression by sequestering the TOX4-NTSR1 signaling axis
Source: J Biomed Sci. 2023 Sep 19;30:80. doi: 10.1186/s12929-023-00973-9 (PMC10507979; doi:10.1186/s12929-023-00973-9)
Supplement: Supplementary file 8 — Additional file 8: Figure S1. SLCO4A1-AS1 overexpression inhibits cancer stem cells properties. A Stable overexpression of SLCO4A1-AS1 was confirmed by RT-qPCR. The data of RT-qPCR are presented as mean ± SD (***p < 0.001). B Morphology and C average number of spheres per well of control and SLCO4A1-AS1-overexpressing lung cancer cells were calculated in the sphere formation assay (**p < 0.01, ***p < 0.001). Scale bar = 50 µm. D Overexpression of SLCO4A1-AS1 decreased the proportion of ALDH+ lung cancer cells using the Aldefluor™ assay (**p < 0.01, ***p < 0.001). DEAB is an ALDH inhibitor used to improve assay specificity. E RT-qPCR was performed to measure the expression levels of ALDH1A1 in SLCO4A1-AS1-overexpressing lung cancer cells. RT-qPCR data are presented as mean ± SD (*p < 0.05, **p < 0.01, ***p < 0.001). F The SLCO4A1-AS1-overexpressing cells and control cells were stained with Hoechst 33342 and then the side population is gated and distinguished by the treatment of verapamil (vera). The results from four independent experiments were quantified on the right, with statistical significance (***p < 0.001) observed. Figure S2. The effects of overexpressing SLCO4A1-AS1 on cell proliferation and drug resistance. A Cell growth was assessed using the CCK-8 assay in SLCO4A1-AS1 expression PC9/gef and H1299 cell lines (ns, not statistically significant). B CCK-8 assay for SLCO4A1-AS1 expressing PC9/gef and H1299 cells treated with increasing concentrations of osimertinib and paclitaxel for 96 hours, respectively (ns, not statistically significant).Figure S3. SLCO4A1-AS1 suppresses cell motility. A The effect of SLCO4A1-AS1-overexpressing CL1-5 cells on migration and invasion was performed using transwell and matrigel assays, respectively. Quantification of migratory and invasive cell numbers are shown (*p < 0.05; ns, not statistically significant). B Immunofluorescence staining for p-FAK (red), p-paxillin, (red), rhodamine-phalloidin for F-actin (red), and nuclei ( [file 12929_2023_973_MOESM8_ESM.pdf]

# Additional Figures

## Fig. S1

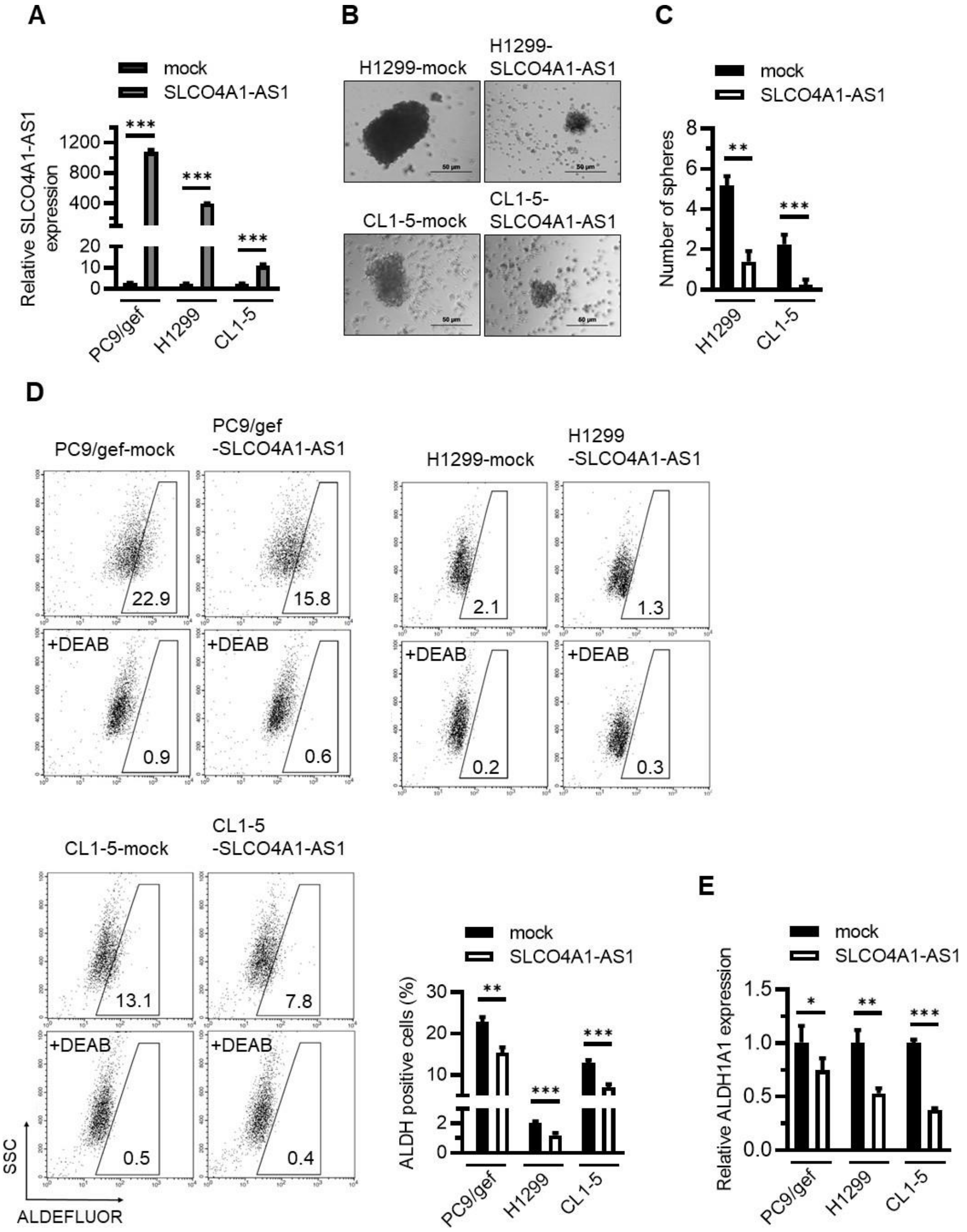


(*Continued*)

**Fig. S1 (*Continued*)**

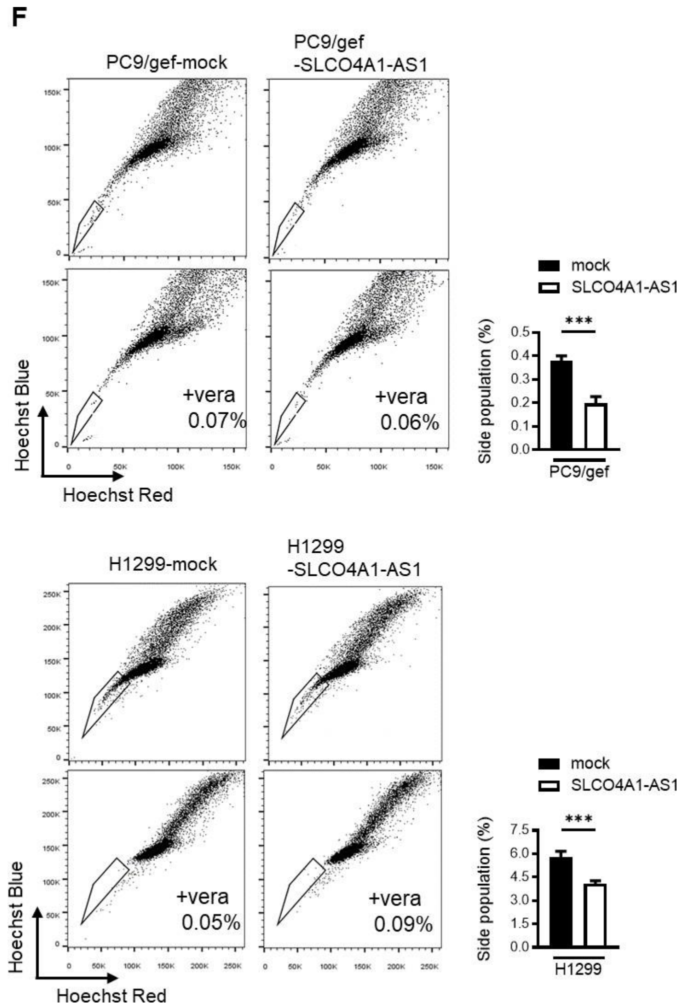


**Fig. S1 SLCO4A1-AS1 overexpression inhibits cancer stem cells properties. A** Stable overexpression of SLCO4A1-AS1 was confirmed by RT-qPCR. The data of RT-qPCR are presented as mean ± SD (***$p < 0.001$). **B** Morphology and **C** average number of spheres per well of control and SLCO4A1-AS1-overexpressing lung cancer cells were calculated in the sphere formation assay (**$p < 0.01$, ***$p < 0.001$). Scale bar = 50 µm. **D** Overexpression of SLCO4A1-AS1 decreased the proportion of $ALDH^+$ lung cancer cells using the Aldefluor™ assay (**$p < 0.01$, ***$p < 0.001$). DEAB is an ALDH inhibitor used to improve assay specificity. **E** RT-qPCR was performed to measure the expression levels of ALDH1A1 in SLCO4A1-AS1-overexpressing lung cancer cells. RT-qPCR data are presented as mean ± SD (*$p < 0.05$, **$p < 0.01$, ***$p$ <

0.001). **F** The SLCO4A1-AS1-overexpressing cells and control cells were stained with Hoechst 33342 and then the side population is gated and distinguished by the treatment of verapamil (vera). The results from four independent experiments were quantified on the right, with statistical significance (****p* < 0.001) observed.

**Fig. S2**

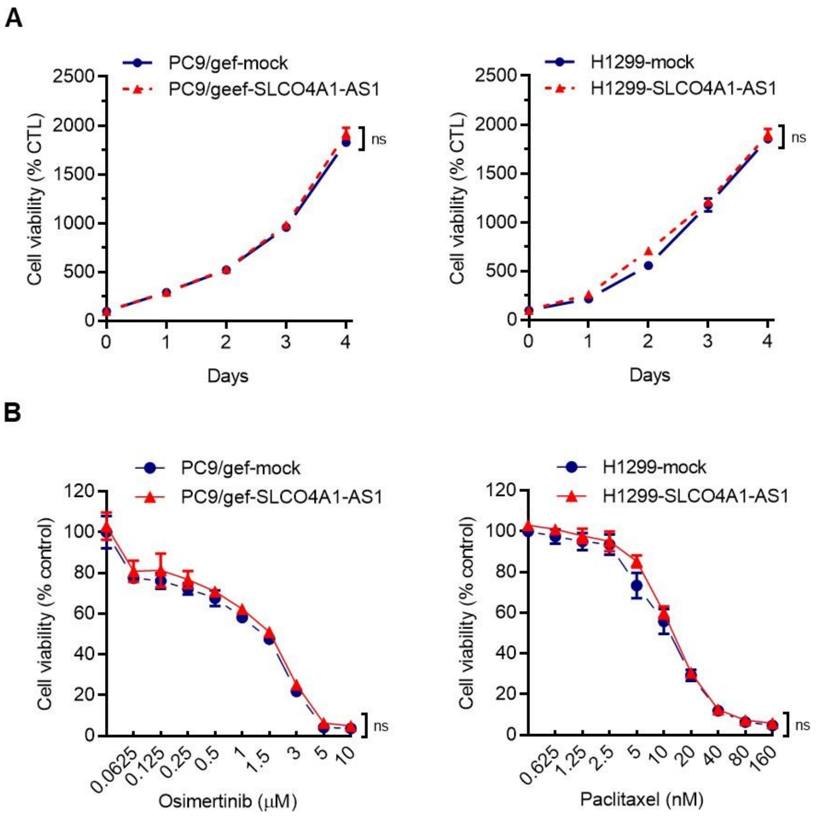


**Fig. S2 The effects of overexpressing SLCO4A1-AS1 on cell proliferation and drug resistance. A** Cell growth was assessed using the CCK-8 assay in SLCO4A1-AS1 expression PC9/gef and H1299 cell lines (ns, not statistically significant). **B** CCK-8 assay for SLCO4A1-AS1 expressing PC9/gef and H1299 cells treated with increasing concentrations of osimertinib and paclitaxel for 96 hours, respectively (ns, not statistically significant).

**Fig. S3**

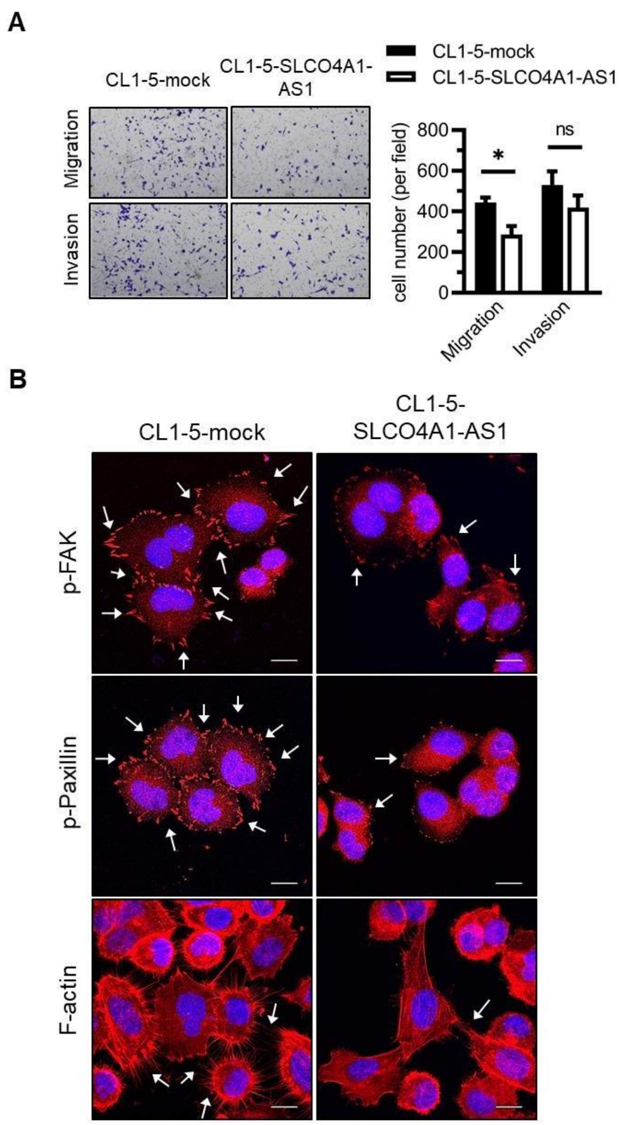


**Fig. S3 SLCO4A1-AS1 suppresses cell motility. A** The effect of SLCO4A1-AS1-overexpressing CL1-5 cells on migration and invasion was performed using transwell and matrigel assays, respectively. Quantification of migratory and invasive cell numbers are shown (*$p < 0.05$; ns, not statistically significant). **B** Immunofluorescence staining for p-FAK (red), p-paxillin, (red), rhodamine-phalloidin for F-actin (red), and nuclei (DAPI, blue) in the control and SLCO4A1-AS1-overexpressing CL1-5 cells, indicated by white arrows and scale bar = 20 µm.

**Fig. S4**

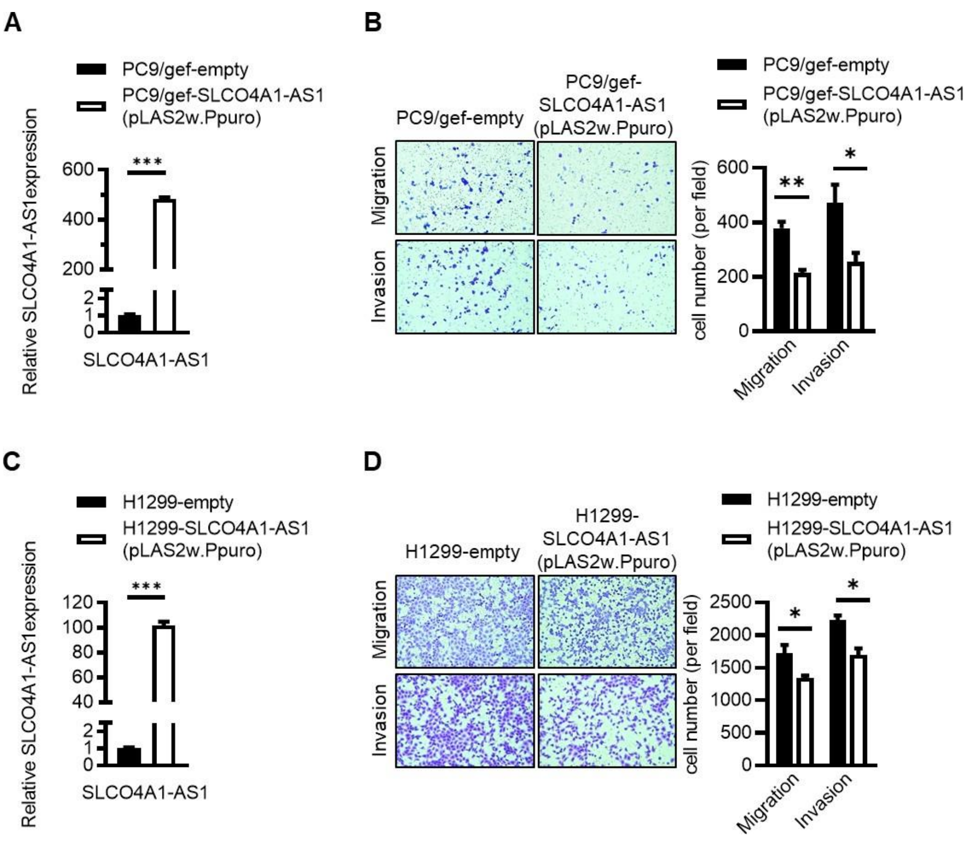


**Fig. S4 The transient expression of SLCO4A1-AS1 inhibits lung cancer cells migration and invasion.** Transient expression of SLCO4A1-AS1 was confirmed in **A** PC9/gef and **C** H1299 cells. These data of RT-qPCR are presented as mean ± SD (***$p$ < 0.001). The effect of SLCO4A1-AS1 transient expression on migration and invasion of **B** PC9/gef and **D** H1299 cells was evaluated. Quantification of migratory and invasive cell numbers are shown (*$p$ < 0.05, **$p$ < 0.01).

**Fig. S5**

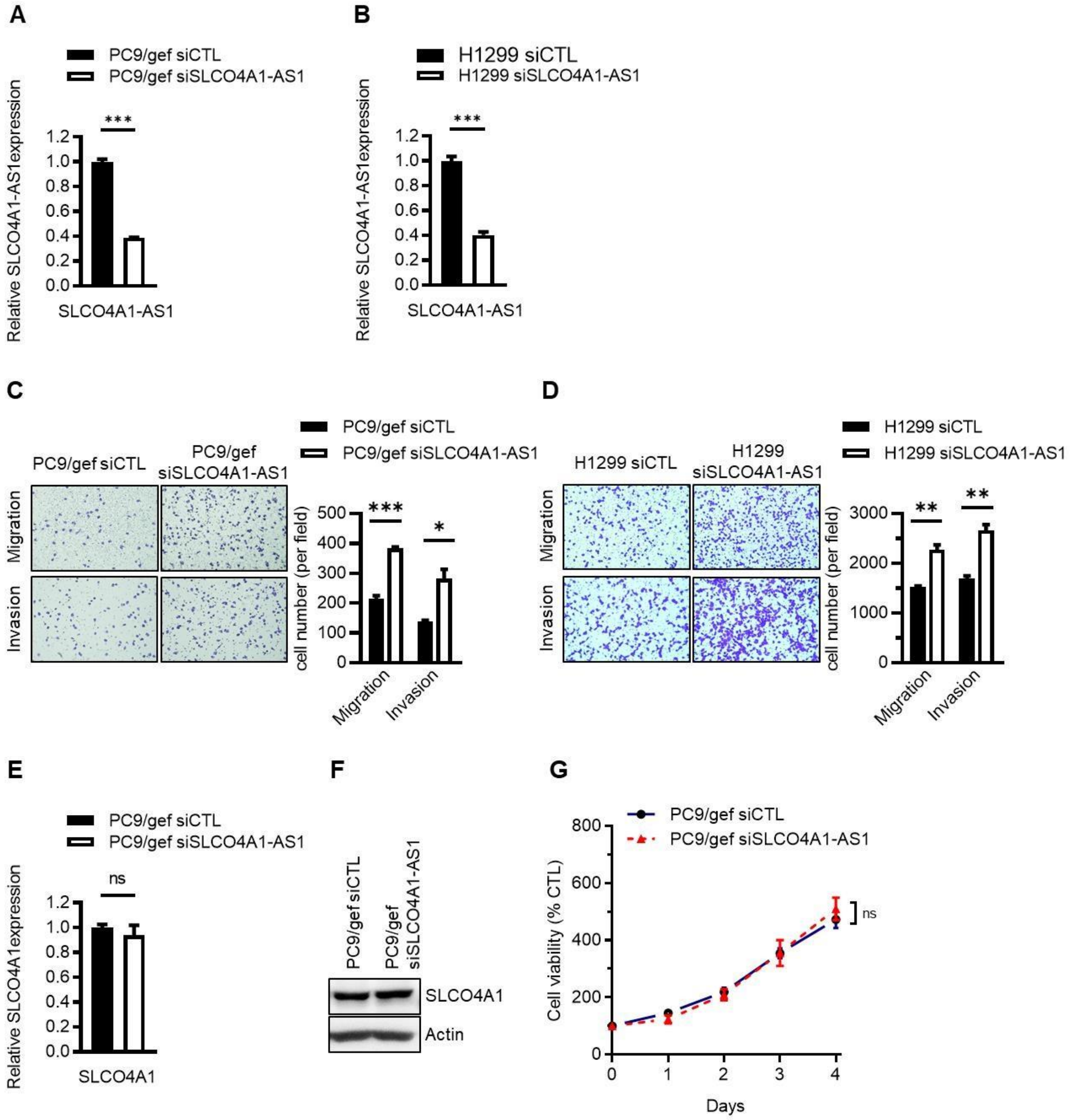


**Fig. S5 Knockdown of SLCO4A1-AS1 promotes lung cancer cell migration and invasion.** The efficiency of knockdown SLCO4A1-AS1 in **A** PC9/gef cells and **B** H1299 through siRNA transfection was confirmed by RT-qPCR. The data of RT-qPCR are presented as mean ± SD (***$p$ < 0.001). The effect of SLCO4A1-AS1 knockdown on migration and invasion of **C** PC9/gef and **D** H1299 cells was measured using transwell and matrigel assays, respectively. Quantification of migratory and invasive

cell numbers are shown (*$p < 0.05$, **$p < 0.01$, ***$p < 0.001$). **E** The effect of SLCO4A1-AS1 knockdown on *SLCO4A1* mRNA expression was evaluated by RT-qPCR (ns, not statistically significant). **F** The impact of SLCO4A1-AS1 knockdown on SLCO4A1 protein levels was assessed by western blotting assay. **G** Cell growth in SLCO4A1-AS1-silenced PC9/gef cells was assessed using the CCK-8 assay (ns, not statistically significant).

**Fig. S6**

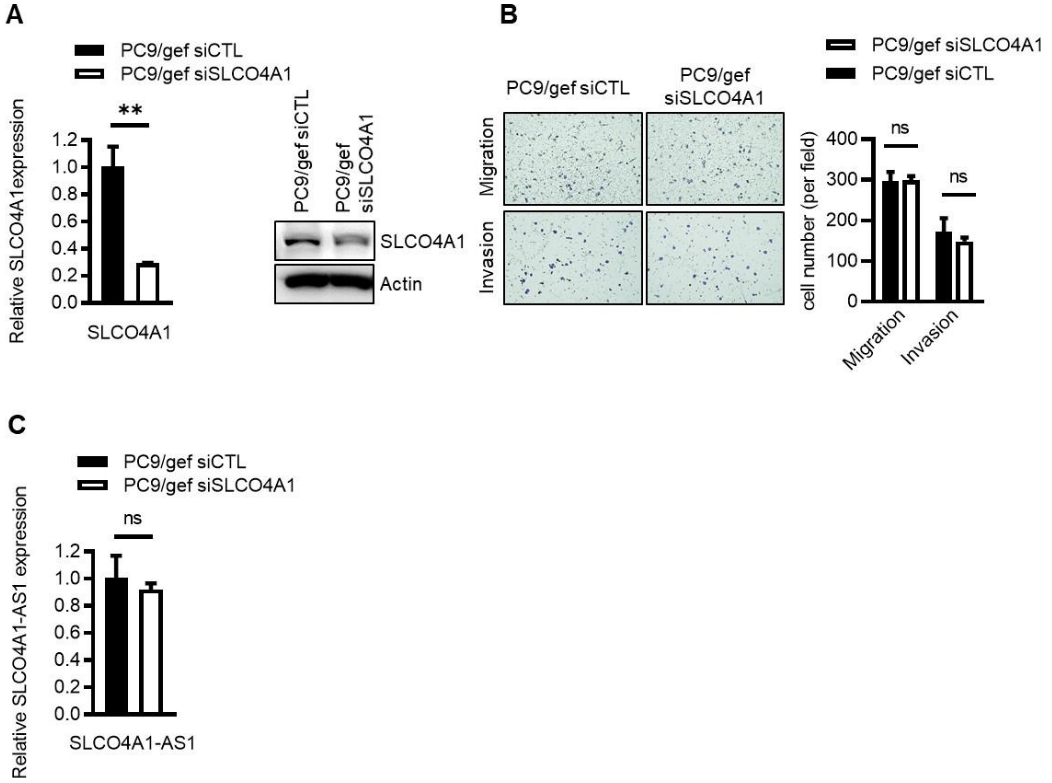


**Fig. S6 Knockdown of SLCO4A1 did not affect the migration and invasion abilities of PC9/gef cells. A** The efficiency of knockdown SLCO4A1 in PC9/gef cells through siRNA transfection was confirmed by RT-qPCR and western blotting. The data of RT-qPCR are presented as mean ± SD (**$p$ < 0.01). **B** The effect of SLCO4A1 knockdown on migration and invasion of PC9/gef cells was measured using transwell and matrigel assays, respectively. Quantification of migratory and invasive cell numbers are shown (ns, not statistically significant). **C** The effect of SLCO4A1 knockdown on lncRNA SLCO4A1-AS1 expression was evaluated by RT-qPCR (ns, not statistically significant).

**Fig. S7**

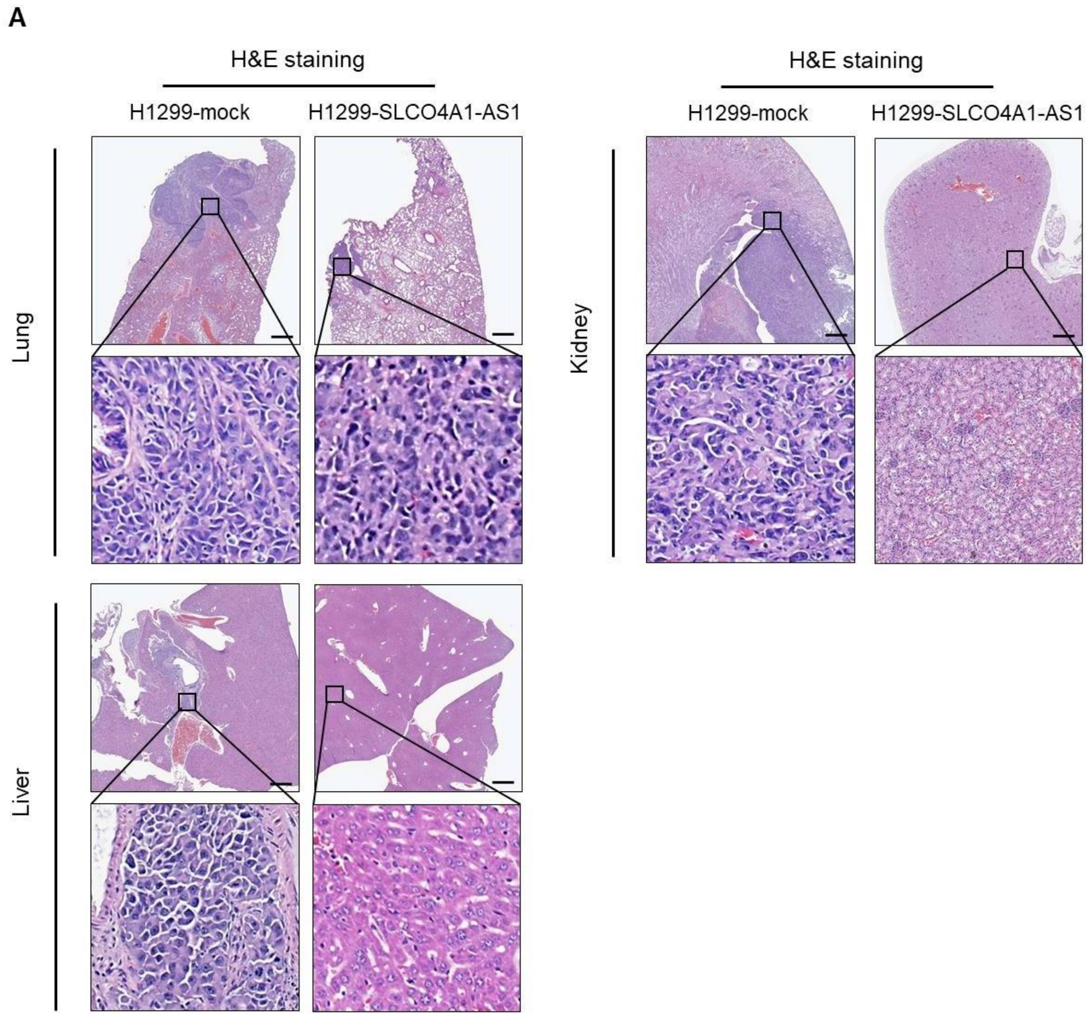


**Fig. S7 SLCO4A1-AS1 inhibits metastases in vivo. A** Representative H&E staining images of lung, kidney, and liver tissue sections from NOD/SCID mice injected with H1299-mock (n=15) or H1299-SLCO4A1-AS1 (n=15), with a scale of 500 μm.

**Fig. S8**

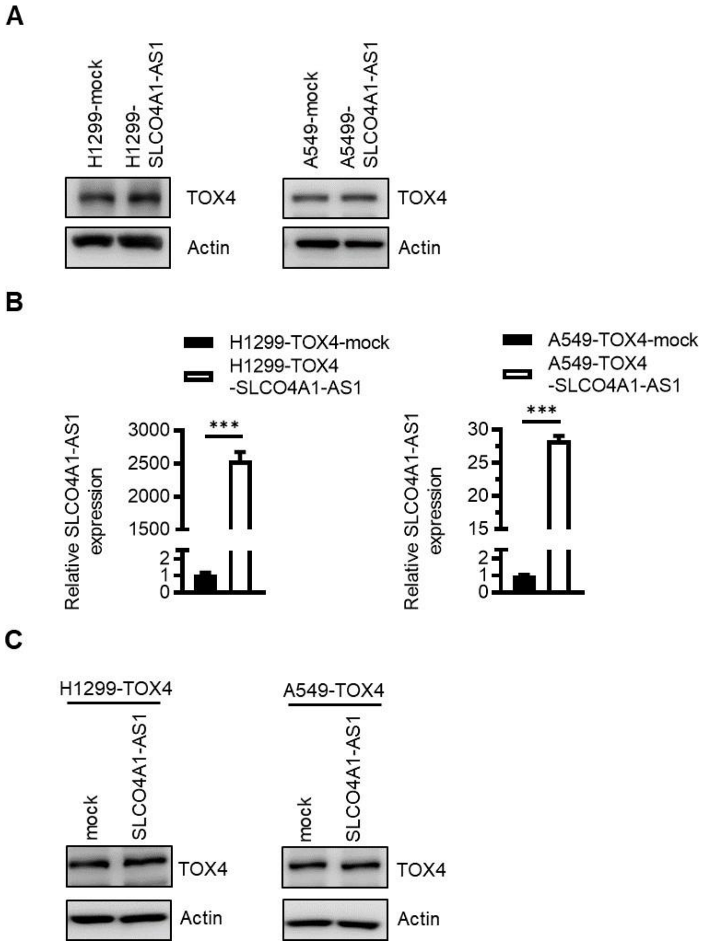


**Fig. S8 SLCO4A1-AS1 did not affect the TOX4 expression levels. A** TOX4 protein expression was detected in SLCO4A1-AS1-expressing H1299 and A549 cells by western blotting. **B** SLCO4A1-AS1 overexpression in H1299-TOX4 and A549-TOX4 cells was evaluated by RT-qPCR assay. The data of RT-qPCR are presented as mean ± SD (****p* < 0.001). **C** The TOX4 protein levels in SLCO4A1-AS1-expressing H1299-TOX4 and A549-TOX4 cells were measured by western blotting.

**Fig. S9**

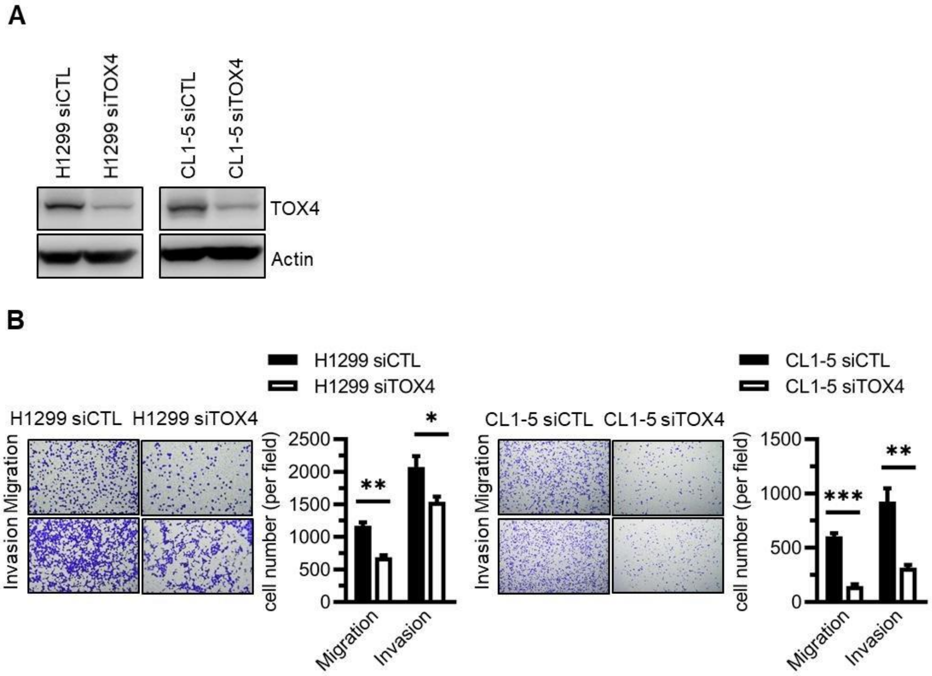


**Fig. S9 TOX4 knockdown inhibits lung cancer cell migration and invasion. A** H1299 and CL1-5 cells were transfected with TOX4 small interfering RNAs (siTOX4) or scramble siRNA (siCTL). The effect of siRNAs was evaluated by western blotting. **B** The effect of TOX4 knockdown on H1299 and CL1-5 cell migration and invasion were measured using transwell and matrigel assays, respectively. Quantification of migratory and invasive cell numbers are shown (*$p < 0.05$, **$p < 0.01$, ***$p < 0.001$).

**Fig. S10**

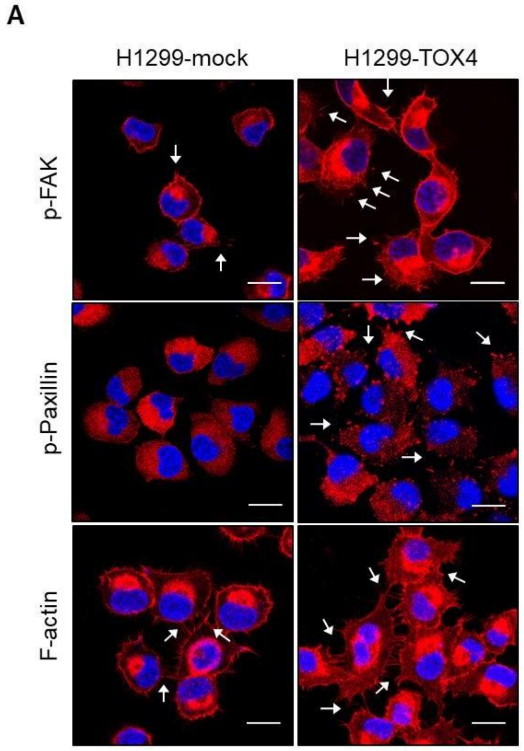


**Fig. S10 TOX4 promotes cell motility. A** Immunofluorescence staining for p-FAK (red), p-paxillin, (red), rhodamine-phalloidin for F-actin (red), and nuclei (DAPI, blue) in the control and TOX4-overexpressing H1299 cells, indicated by white arrows and scale bar = 20 µm.

**Fig. S11**

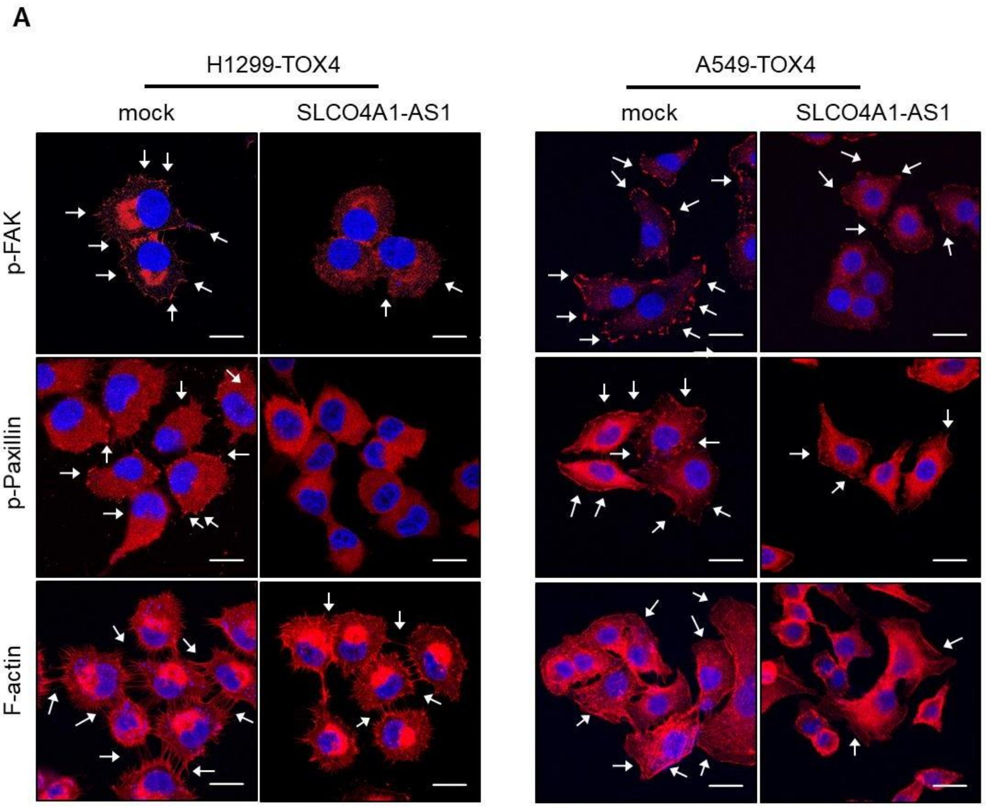


**Fig. S11 SLCO4A1-AS1 rescues the TOX4-promoted cell motility. A** Immunofluorescence staining for p-FAK (red), p-paxillin, (red), rhodamine-phalloidin for F-actin (red), and nuclei (DAPI, blue) in the control and SLCO4A1-AS1-expressing H1299-TOX4 and A549-TOX4 cells, indicated by white arrows and scale bar = 20 μm.

**Fig. S12**

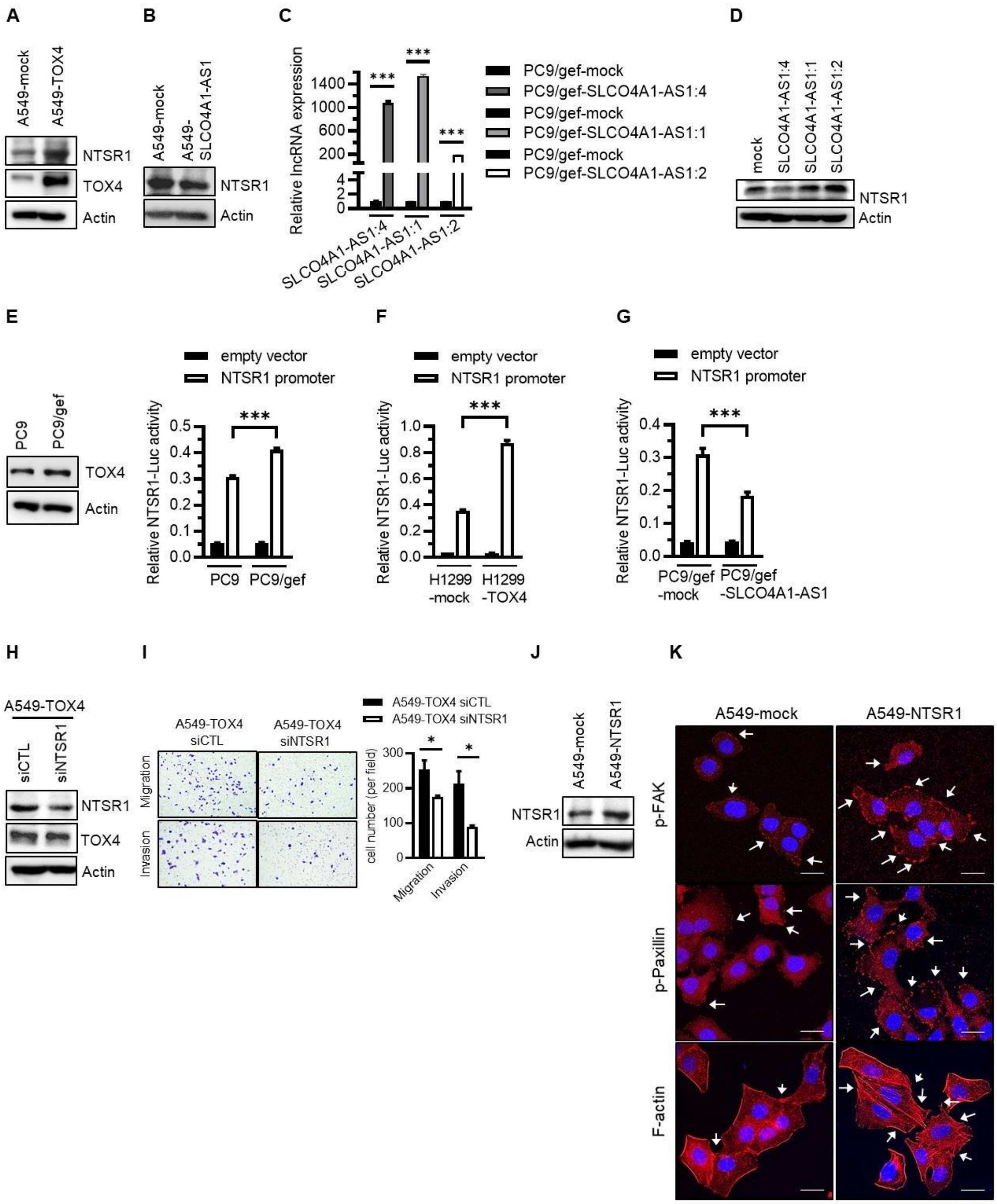


**Fig. S12** ***NTSR1*** **is the SLCO4A1-AS1/TOX4 downstream target. A** NTSR1 protein expression was detected in TOX4-expressing A549 cells by western blotting. **B** The protein expression of NTSR1 was detected in SLCO4A1-AS1-expressing A549 cells using western blotting. **C** The expression level of SLCO4A1-AS1 transcripts were

evaluated with transcript specific primers by RT-qPCR assay. **D** The protein expression level of NTSR1 were determined in different SLCO4A1-AS1 transcript-expressing PC9/gef cells. **E** Western blotting was used to detect TOX4 and *NTSR1* promoter luciferase assay was performed in PC9 and PC9/gef cells after transfection of pGL3/NTSR1 luciferase vector or pGL3 empty vector. **F** *NTSR1* promoter luciferase assay was done by transfecting H1299-mock and H1299-TOX4 cells with pGL3/NTSR1 luciferase vector or pGL3 empty vector. **G** *NTSR1* promoter luciferase assay was done by transfecting PC9/gef-mock and PC9/gef-SLCO4A1-AS1 cells with pGL3/NTSR1 luciferase vector or pGL3 empty vector. All the relative luciferase activities are presented as means ± SD (***$p < 0.001$). **H** A549-TOX4 cells were transfected with NTSR1 small interfering RNAs (siNTSR1) or scramble siRNA (siCTL). The effect of siRNAs was evaluated by western blotting. **I** The effect of NTSR1 knockdown on A549-TOX4 cell migration and invasion were measured using transwell and matrigel assays, respectively. Quantification of migratory and invasive cell numbers are shown (*$p < 0.05$). **J** Stable overexpression of NTSR1 in A549 was evaluated by western blotting. **K** Immunofluorescence staining for p-FAK (red), p-paxillin, (red), rhodamine-phalloidin for F-actin (red), and nuclei (DAPI, blue) in the control and A549-NTSR1 cells, indicated by white arrows and scale bar = 20 μm.

## Fig. S13 refer to Fig. 5

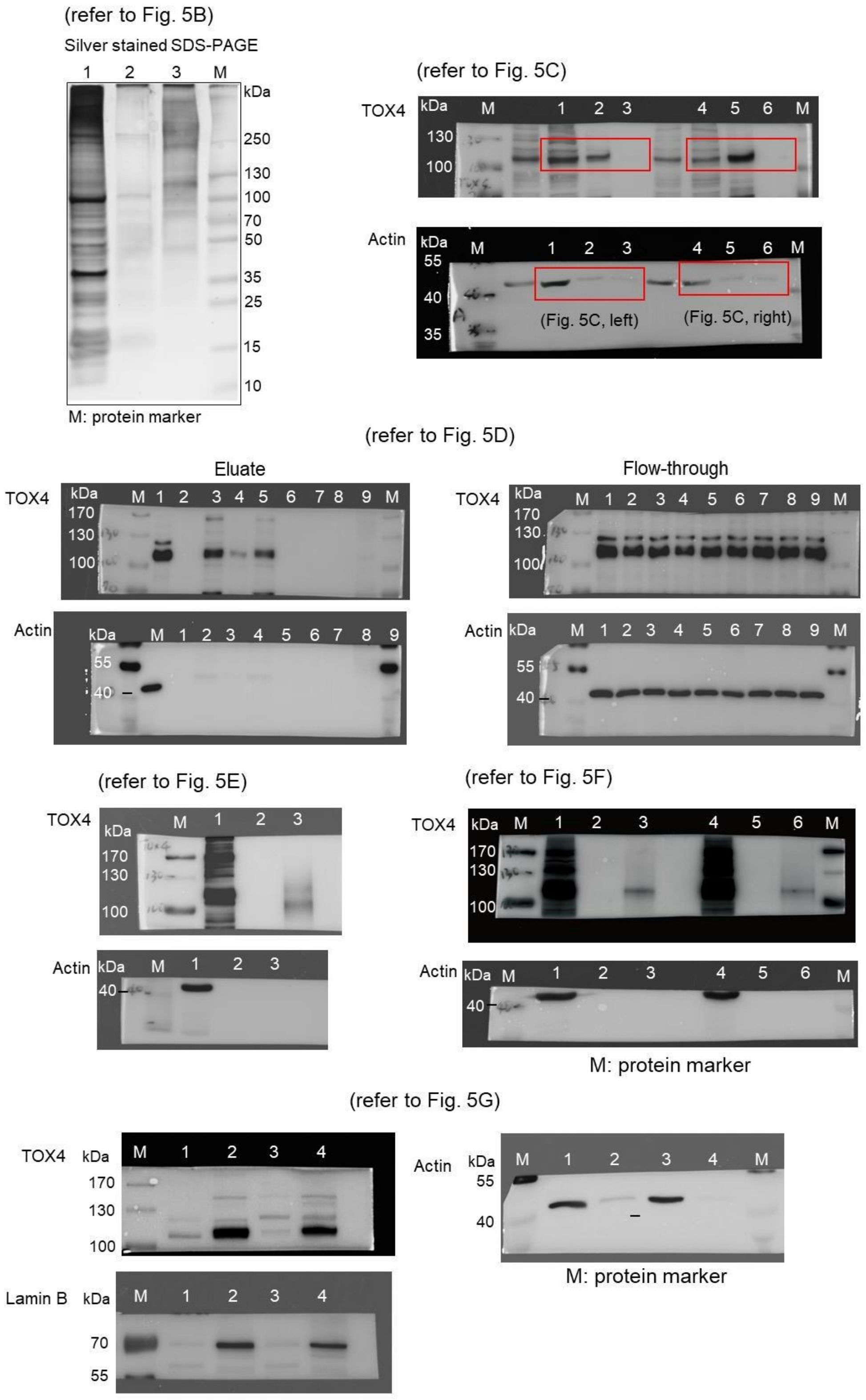


**Fig. S13 Original films refer to Fig. 5B-5G.**

## Fig. S14 refer to Fig. 6

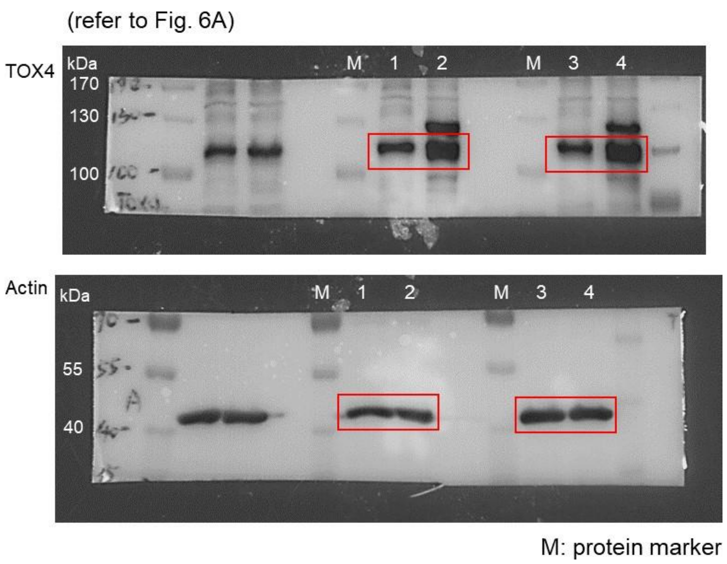


**Fig. S14 Original films refer to Fig. 6A.**

## Fig. S15 refer to Fig. 7

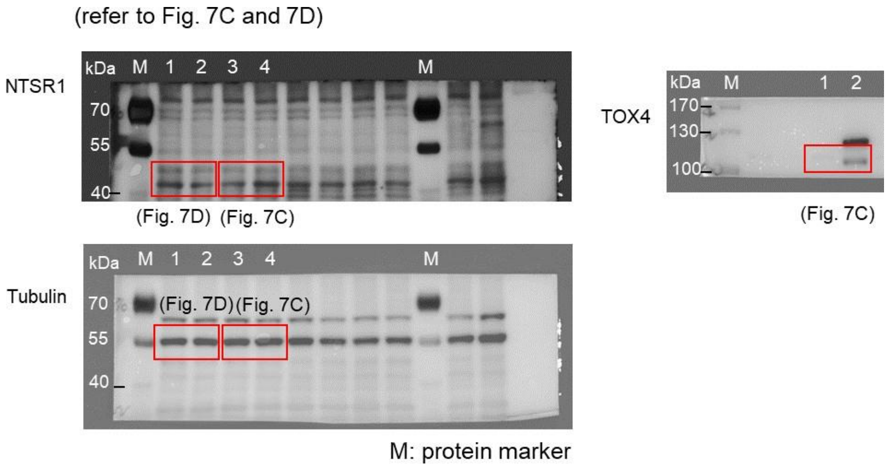


**Fig. S15 Original films refer to Fig. 7C and 7D.**

**Fig. S16 refer to Fig. 8**

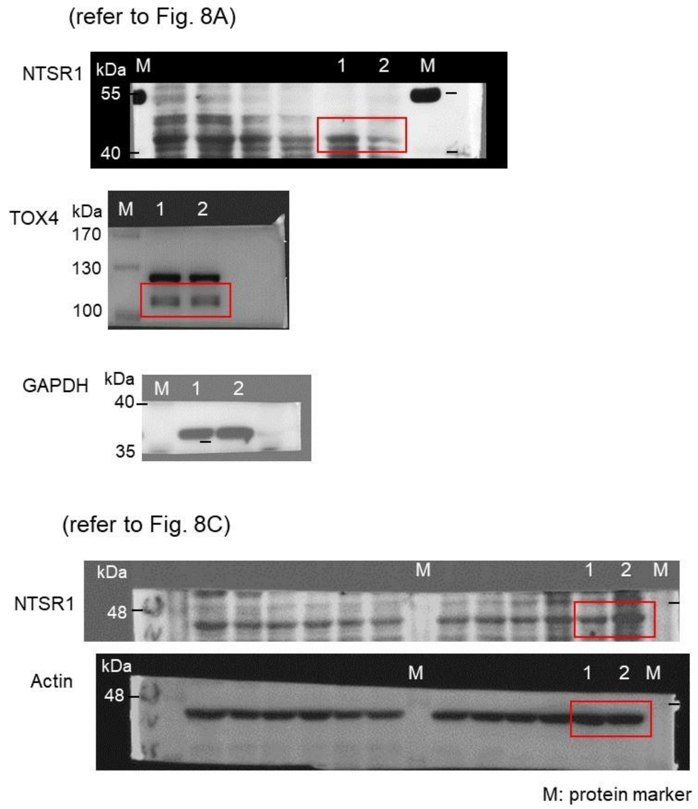


**Fig. S16 Original films refer to Fig. 8A and 8C.**

**Fig. S17**

**A**

PC9

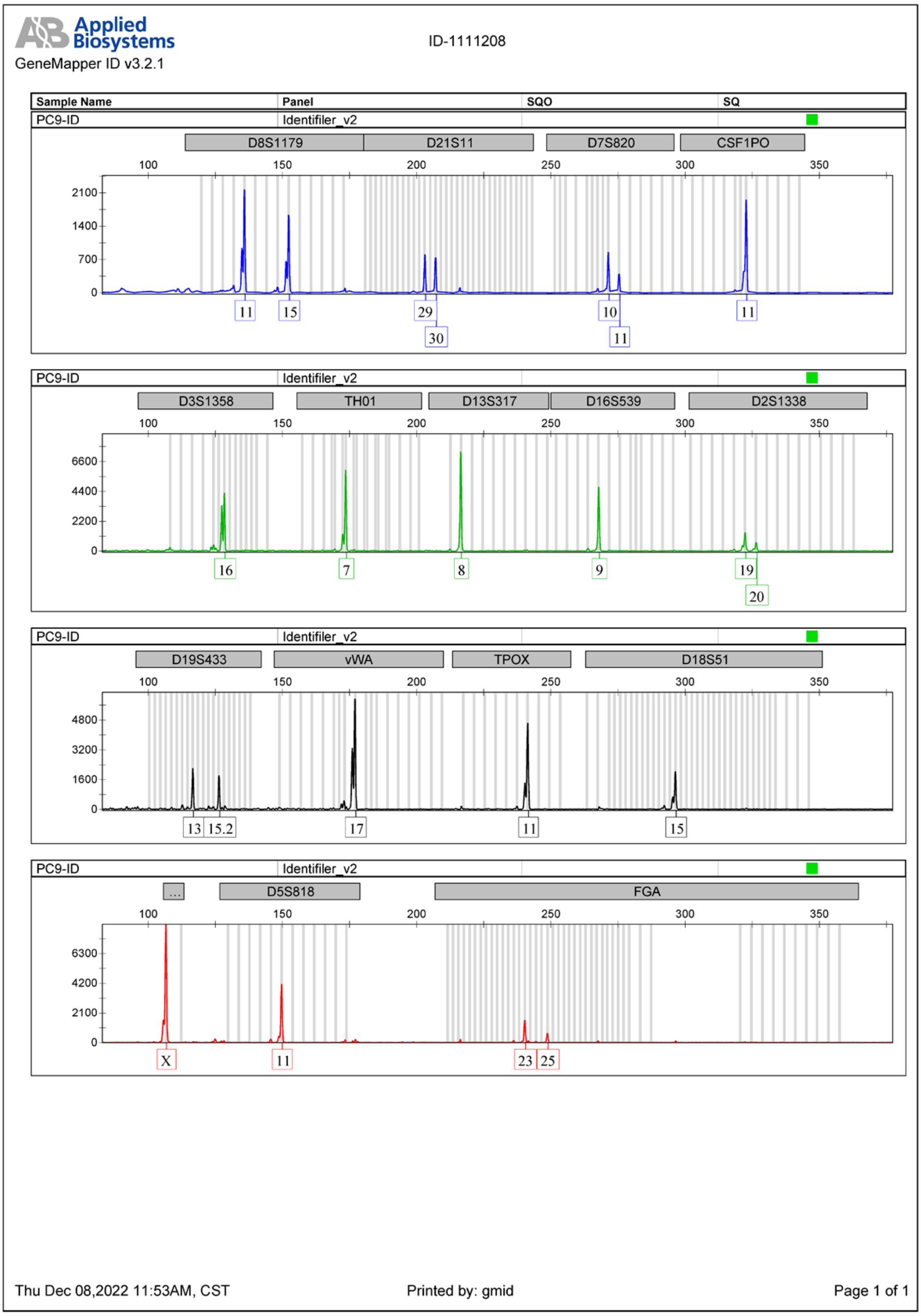


**(*Continued*)**

**B (*Continued*)**

PC9/gef

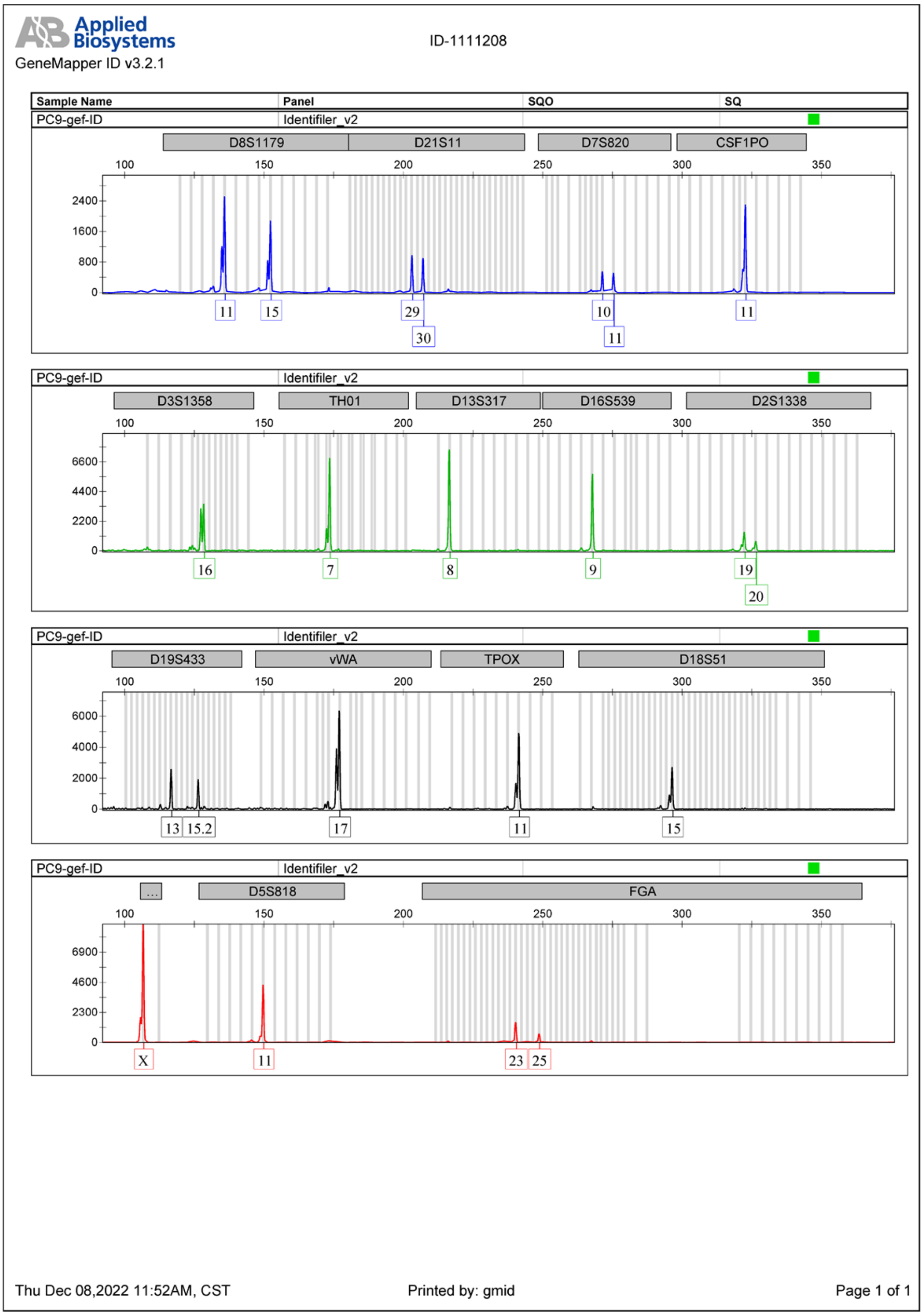


**(*Continued*)**

**C (*Continued*)**

H1299

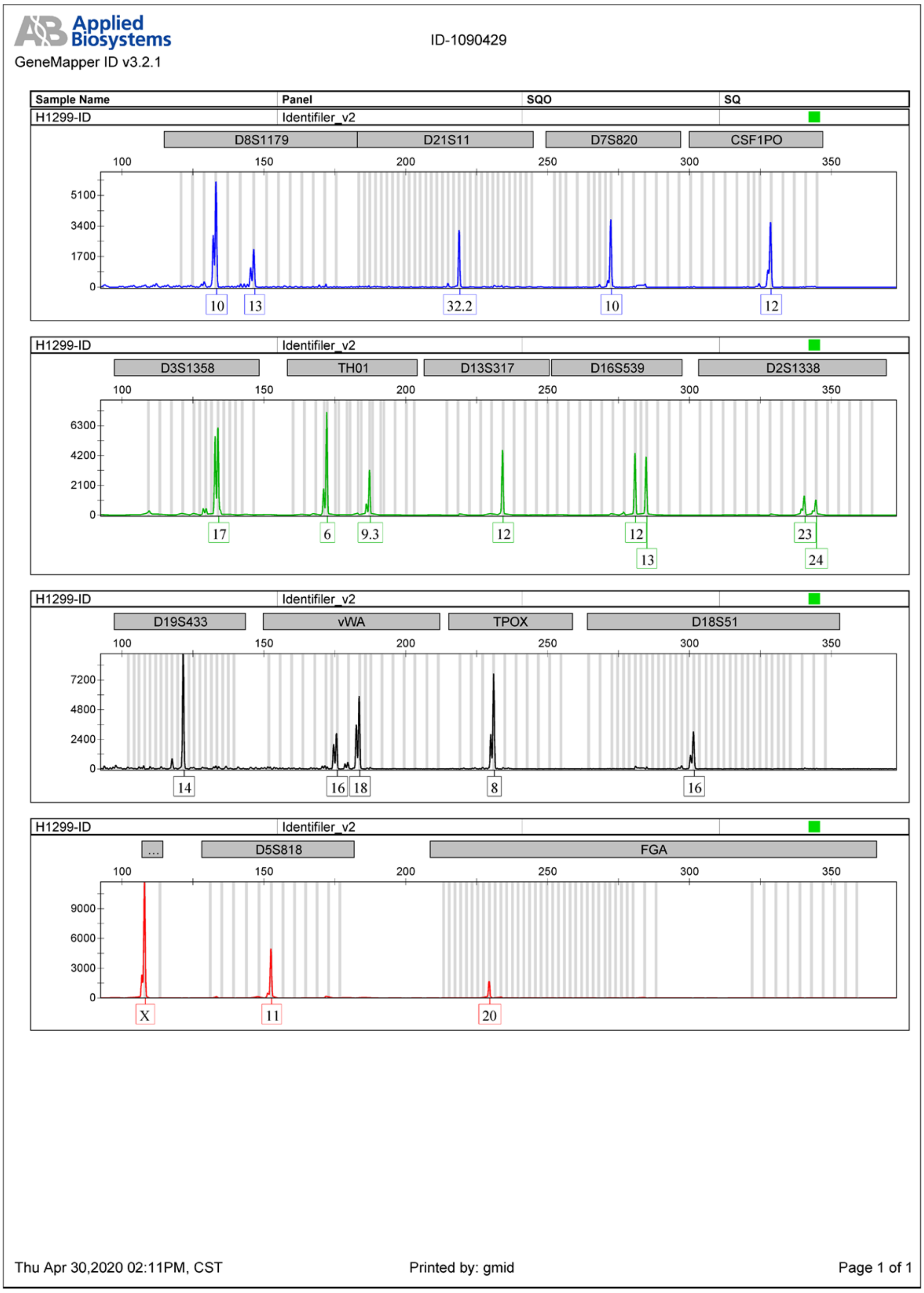


**(*Continued*)**

**D (*Continued*)**

A549

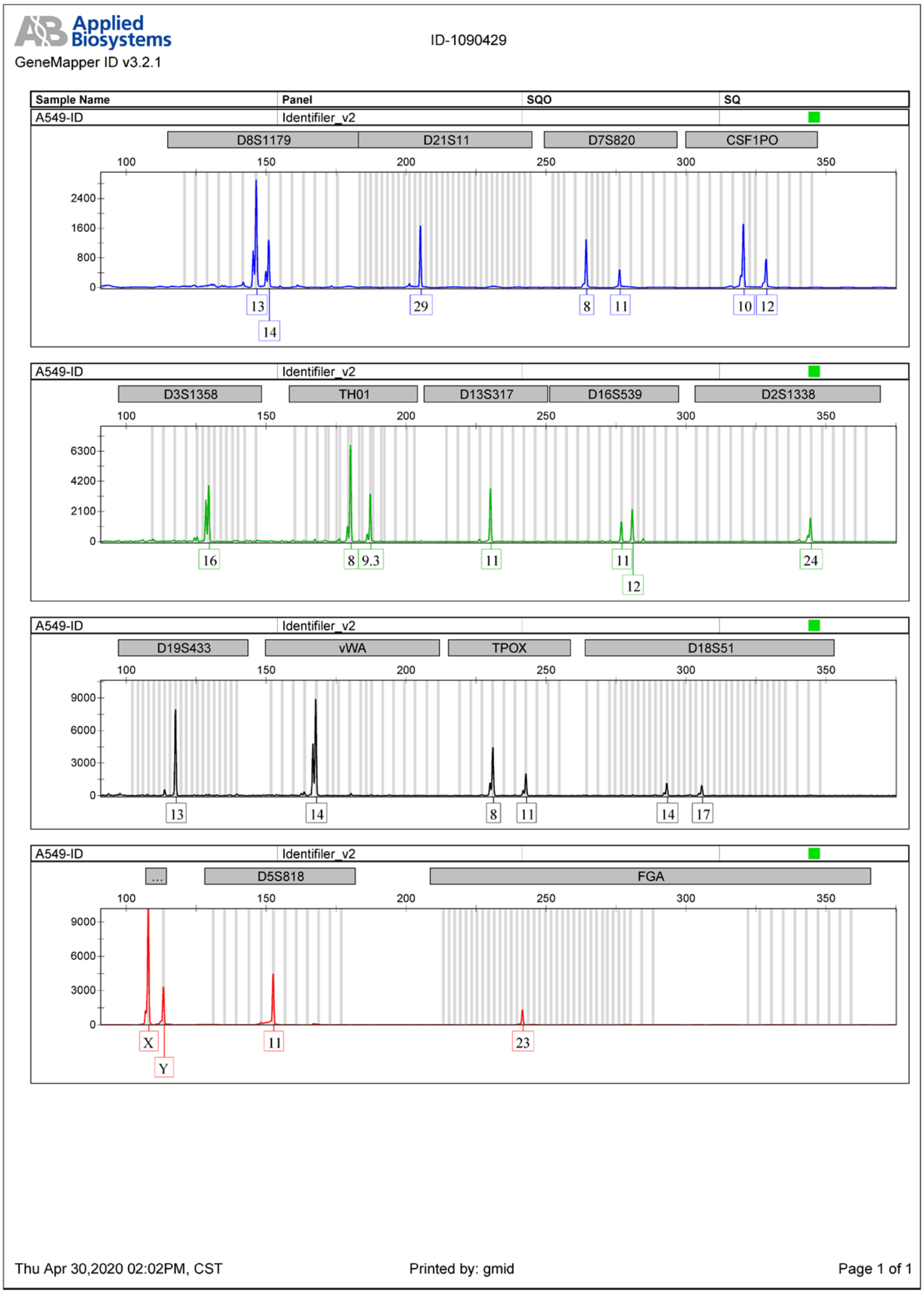


**(*Continued*)**

**E (*Continued*)**

CL1-5

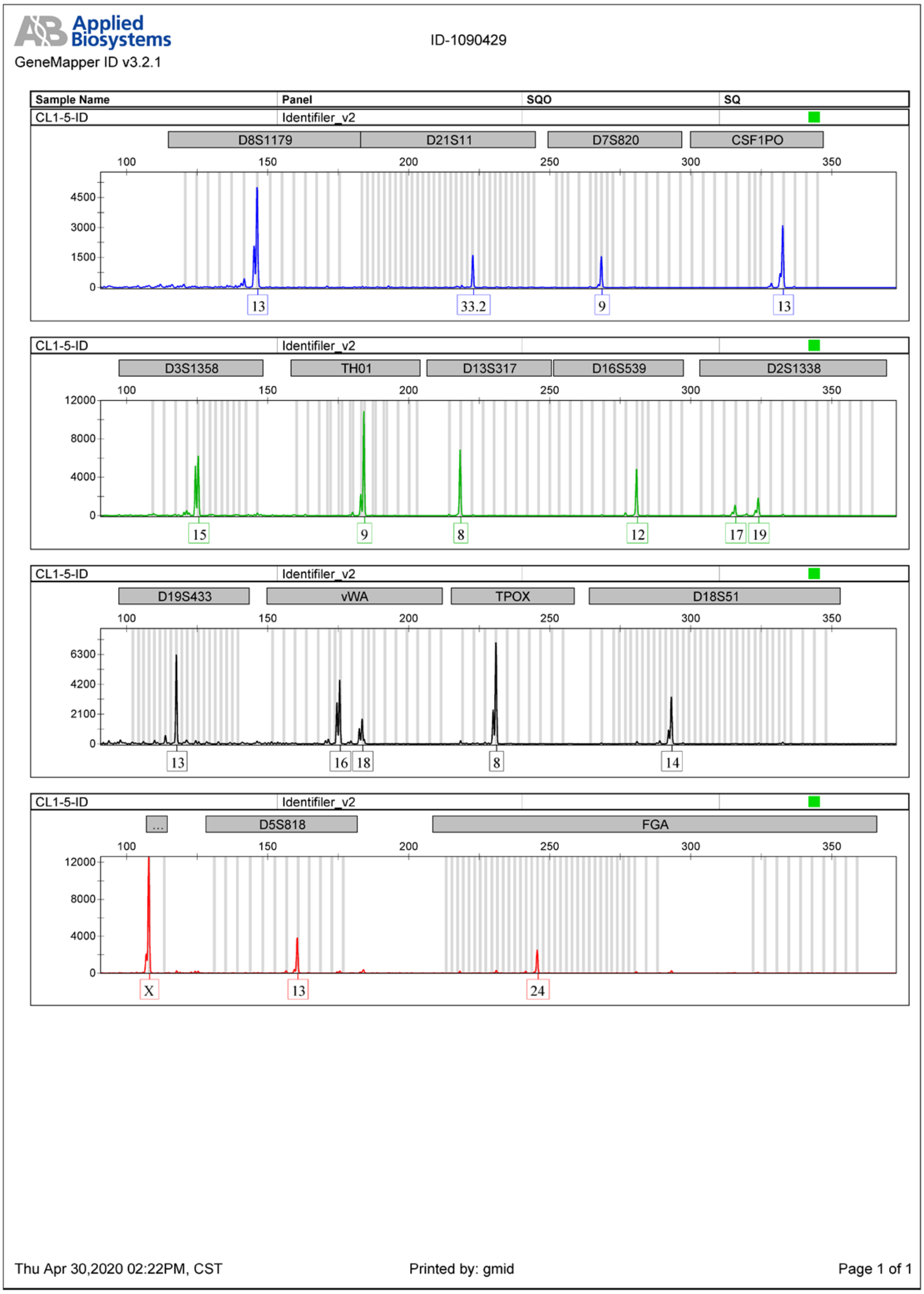


**(*Continued*)**

**F (*Continued*)**

CL1-0

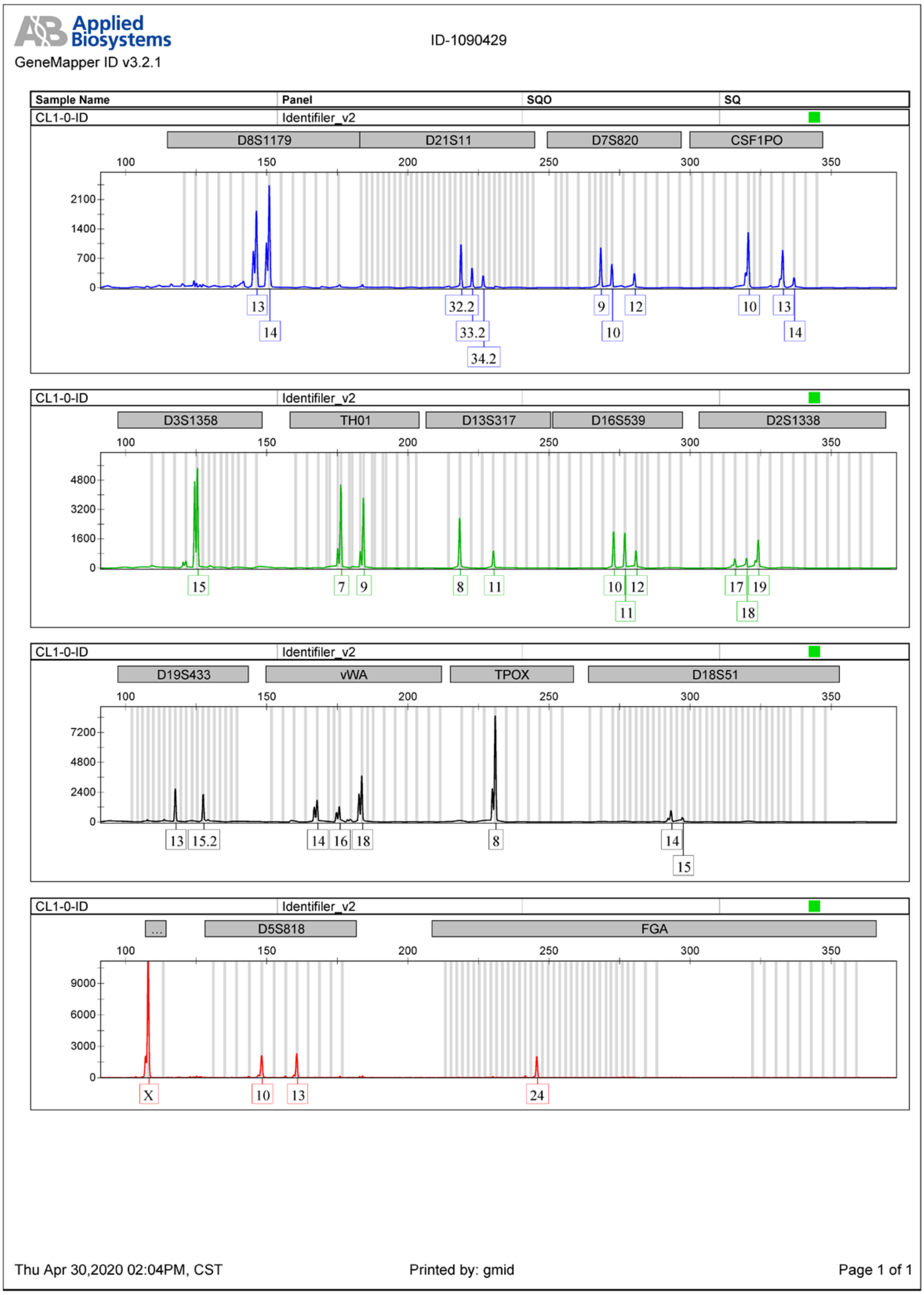


**Fig. S17 STR DNA profile of lung cancer cell lines. A** PC9, **B** PC9/gef, **C** H1299, **D** A549, **E** CL1-5, and **F** CL1-0.
