# Supplementary material for: LncRNA SLCO4A1-AS1 suppresses lung cancer progression by sequestering the TOX4-NTSR1 signaling axis
Source: J Biomed Sci. 2023 Sep 19;30:80. doi: 10.1186/s12929-023-00973-9 (PMC10507979; doi:10.1186/s12929-023-00973-9)
Supplement: Supplementary file 9 — Additional file 9. Additional methods. [file 12929_2023_973_MOESM9_ESM.pdf]

Additional Methods

Cell proliferation assays

Cell proliferative ability was measured using CCK-8. Cells were seeded into a 96-well plate and 10 µL of CCK-8 solution was added to each well at indicated time points (0, 24, 48, 72, and 96 hours) for 2hr at 37 °C. Afterwards, absorbance was determined at a wavelength of 450 nm using a microplate reader (Molecular Devices, San Jose, CA, USA).

Spheres formation

Cells were seeded in DMEM/F12 (Thermo Fisher Scientific, Waltham, MA, USA), supplemented with 1% penicillin/streptomycin (Thermo Fisher) and 1% Insulin-Transferrin-Selenium-Ethanolamine (ITS) (Merck, St. Louis, MO, USA), 20 ng /mL EGF (PeproTech, Boston, MA, USA), and 25 ng/mL bFGF (PeproTech), in ultra-low attachment 24-well plates (Corning Life Sciences, Oneonta, NY, USA). The plates were incubated in a humidified atmosphere of 5% CO_2 at 37 °C. The cells were cultured for 14-21 days and the spheres large than 50 µm were counted and imaged.

Side population

Cells were resuspended at a concentration of 1×10^6 cells/mL in pre-warmed PBS containing 2% FBS. The cells were then labelled with 5µg/mL Hoechst 33342 dye (Sigma-Aldrich; St. Louis, MO, USA) at a final concentration of 5 µg/mL in the presence or absence of verapamil (150 µM; Sigma-Aldrich) and incubation at 37°C for 90 min. After incubation, the cells were washed with ice-cold 2% FBS/PBS, centrifuged at 4°C, and resuspended in ice-cold 2% FBS/PBS. To gate viable cells, propidium iodide (PI; BD Biosciences, San Diego, CA, USA) were added to the cells at a final

concentration of 2 μg/mL. Flow cytometric analyses were performed using a BD® LSR II Flow Cytometer (BD Biosciences).

NTSR1 promoter luciferase reporter assay

The promoter sequence (-1000 to 150 from the TSS) of *NTSR1* was synthesized and cloned into pGL3 basic luciferase reporter vector (luciferase pGL3/NTSR1 vector) by BIOTOOLS (Xizhi Dist, New Taipei City, Taiwan). Indicated cancer cell lines were transfected with either luciferase pGL3/NTSR1 vector or the luciferase pGL3 empty vector using Lipofectamine 2000 (Thermo Fisher Scientific) according to manufacturer's instructions. After 24 hours of transfection, luciferase activity was measured using a dual-luciferase reporter assay system (Promega; Madison, WI, USA).
